# Progress and Prospects of Mycorrhizal Fungal Diversity in Orchids

**DOI:** 10.3389/fpls.2021.646325

**Published:** 2021-05-07

**Authors:** Taiqiang Li, Wenke Yang, Shimao Wu, Marc-André Selosse, Jiangyun Gao

**Affiliations:** ^1^Yunnan Key Laboratory of Plant Reproductive Adaptation and Evolutionary Ecology, Yunnan University, Kunming, China; ^2^Laboratory of Ecology and Evolutionary Biology, Yunnan University, Kunming, China; ^3^Institut de Systématique, Évolution, Biodiversité, UMR 7205, CNRS, MNHN, UPMC, EPHE, Muséum National d’Histoire Naturelle, Sorbonne Universités, Paris, France; ^4^Department of Plant Taxonomy and Nature Conservation, Faculty of Biology, University of Gdańsk, Gdańsk, Poland

**Keywords:** orchid mycorrhizal fungi, orchid non-mycorrhizal fungi, primer selection, fungal diversity, mycorrhizal specificity, environmental filtering, spatio-temporal variation

## Abstract

Orchids form mycorrhizal symbioses with fungi in natural habitats that affect their seed germination, protocorm growth, and adult nutrition. An increasing number of studies indicates how orchids gain mineral nutrients and sometime even organic compounds from interactions with orchid mycorrhizal fungi (OMF). Thus, OMF exhibit a high diversity and play a key role in the life cycle of orchids. In recent years, the high-throughput molecular identification of fungi has broadly extended our understanding of OMF diversity, revealing it to be a dynamic outcome co-regulated by environmental filtering, dispersal restrictions, spatiotemporal scales, biogeographic history, as well as the distribution, selection, and phylogenetic spectrum width of host orchids. Most of the results show congruent emerging patterns. Although it is still difficult to extend them to all orchid species or geographical areas, to a certain extent they follow the “everything is everywhere, but the environment selects” rule. This review provides an extensive understanding of the diversity and ecological dynamics of orchid-fungal association. Moreover, it promotes the conservation of resources and the regeneration of rare or endangered orchids. We provide a comprehensive overview, systematically describing six fields of research on orchid-fungal diversity: the research methods of orchid-fungal interactions, the primer selection in high-throughput sequencing, the fungal diversity and specificity in orchids, the difference and adaptability of OMF in different habitats, the comparison of OMF in orchid roots and soil, and the spatiotemporal variation patterns of OMF. Further, we highlight certain shortcomings of current research methodologies and propose perspectives for future studies. This review emphasizes the need for more information on the four main ecological processes: dispersal, selection, ecological drift, and diversification, as well as their interactions, in the study of orchid-fungal interactions and OMF community structure.

## Introduction

Biological interactions are key for the construction and maintenance of communities (Bascompte and Jordano, 2007; Bonfante and Anca, 2009). Fungi interact with the roots of all vascular plants, 85% of them form a mutually symbiotic relationship called a mycorrhiza (van der Heijden et al., 2015) that is pivotal for plant nutrition as well as for soil biology and chemistry (Smith and Read, 2008; Brundrett and Tedersoo, 2018). As they are crucial to ecosystems, mycorrhizal fungi extensively influence plant populations and communities and are divided into four basic types: ectomycorrhizae (ECM), arbuscular mycorrhizae (AM), ericoid mycorrhizae (ErM), and orchid mycorrhizae (OM; Smith and Read, 2008; van der Heijden et al., 2015; Tedersoo et al., 2020). In this mutualistic relationship, plants receive water and mineral nutrients (particularly phosphorus) from the fungi and are protected against biotic and abiotic stresses, while host plants supply carbon from their photosynthesis (Smith and Read, 2008; Köhler et al., 2018; Tedersoo and Bahram, 2019). The strength of mycorrhizal interactions is evidenced by the evolutionary history of fungal symbionts and host plants (Selosse and Tacon, 1998; Hoeksema et al., 2018). Interestingly, OM are considered as more beneficial to orchids than fungi, which rely on other resources as saprotrophs or endophytes of non-orchid roots (Selosse and Martos, 2014). Thus, the interaction between orchids and fungi appears as interdependent asymmetry (Martos et al., 2012; McCormick and Jacquemyn, 2014; McCormick et al., 2018).

Orchids are the second largest flowering plant family after Asteraceae. In addition to being an “ideal model” group for research on the biodiversity and evolution of interactions (Selosse, 2014), this family represents a “flagship” group for the protection of endangered plants globally (Liu et al., 2015a, 2020; Zhang et al., 2017). There are nearly 30,000–35,000 species of orchids worldwide (ca.10% of angiosperms; The Plant List, 2013; Chase et al., 2015). However, more than 50% of these species are concentrated in the tropical areas of the world (Givnish et al., 2016). They reportedly colonized the epiphytic habitat approximately 35 million years ago (Givnish et al., 2015). The ratio of epiphytic to terrestrial orchids in different countries ranges from nearly 1:1 to 5:1 (Givnish et al., 2015; Zhang et al., 2015).

Fungal symbionts are essential as they provide carbon and minerals to the dust-like, reserveless orchid seeds during the early development (Dearnaley et al., 2016). The plant first develops into a spheroid organism that is achlorophyllous in most terrestrial species, called as a protocorm that later develops roots and shoots. The dependency on fungi continues until the adult stage, albeit to different degrees (Smith and Read, 2008; Waterman et al., 2011; Schweiger et al., 2018; Shefferson et al., 2018). Based on their dependence on fungal carbon, adult orchids are classified as (1) autotrophic (AT) that rely on fungi in the early developmental stage but display reduced dependence for carbon as their photosynthetic apparatus develops; (2) mycoheterotrophic (MH) that remain achlorophyllous and are completely dependent on fungal carbon throughout their life; and (3) mixotrophic (MX) orchids that can acquire carbon compounds from photosynthesis and at adulthood (Dearnaley et al., 2012). The existence of truly AT orchids is currently controversed; their isotopic difference from surrounding autotrophs for ^13^C, ^15^N, ^18^O, and ^2^H abundances suggests that they acquire some fungal biomass (Selosse and Martos, 2014; Gebauer et al., 2016; Schiebold et al., 2018). However, the net flow, considering the potential reverse flow from orchid to fungus, remains unknown in most green orchids (referred to here as AT) and the existence of C flow fungus to orchids, smaller than in the reverse direction, is anciently reported (Cameron et al., 2008).

The scientific community has been interested in orchids-fungi biological interactions, owing to pioneering research on the symbiotic germination of orchids by Noël Bernard at the end of nineteenth century (Selosse et al., 2011). The fungus is colonizing germinating seeds *via* trichomes and suspensor cells (Smith and Read, 2008) as well as root velamina and cortical cells at adult stage (Clements, 1988), further producing a large number of intracellular coiled fungal hyphae, known as pelotons and considered as a structure for exchange during their life or even at their death for C transfers to the plant (Rasmussen, 1995; Selosse, 2014; Dearnaley et al., 2016).

In-depth analysis of fungal diversity provides a better understanding of the plant-fungal interaction framework. Early culture-based methods were unable to accurately identify several isolated strains because (i) many species are uncultivable and (ii) taxonomic discrimination between morphologically similar fungal species is difficult, as exemplified by case of *Serendipita vermifera* (Cruz et al., 2011; Weiß et al., 2016; Bajpai et al., 2019). However, the development in molecular ecology through Sanger sequencing and high-throughput sequencing (HTS) technologies in the last decade has substantially improved our understanding of plant microbiota (Müller et al., 2016; Nilsson et al., 2019; Toussaint et al., 2020). Moreover, implementing these technologies to elucidate orchid-fungal interactions provided vital information about the diversity, community structure, patterns, and molecular mechanisms of this symbiosis (Martos et al., 2012; Jacquemyn et al., 2015a; Fochi et al., 2017; Miura et al., 2018; Unruh et al., 2019). Fungal partners of at least 200 genera of Orchidaceae have been identified through the analysis and assessment of orchid-associated fungi under the influence of various biotic and abiotic factors (Ma et al., 2015) such as developmental stages, habitats, or spatiotemporal scales.

To facilitate an extensive understanding of the diversity and ecological dynamics of orchid-fungal associates and to promote the resource conservation and regeneration of rare or endangered orchids with high research or commercial potential, this article summarizes six research modules: the research methods of orchid-fungal diversity, the primer selection in HTS, the fungal diversity and specificity in orchids, the difference and adaptability of orchid mycorrhizal fungi (OMF) in different habitats, the comparison of OMF in orchid roots and soil, and the spatiotemporal variation patterns of OMF. Thus, this review provides avenues for in-depth insight into this field.

## Research Methods of Orchid-Fungal Diversity

The primary research methods of orchid-fungal diversity include pure culture (dependent on isolation) and molecular identification (independent of isolation; Dearnaley et al., 2012; Kohout et al., 2013; Zettler and Corey, 2018). In fact, orchid mycorrhizal symbionts may be considered as an easy symbiotic system under experimental conditions, since both partners can be cultured aseptically in many cases (Dearnaley et al., 2012). Previous studies have demonstrated the importance of *in vitro* fungal isolation in the understanding and experimenting on OM. Moreover, the identifications of some relatively easy-to-isolate strains and biological effect detection have been reported in recent years (Meng et al., 2019a,b,c; Bell et al., 2020; Gao et al., 2020b; Zhang et al., 2020). Isolation methods mainly include isolation from whole tissue or tissue section, *in situ* seeding and trapping isolation, and single peloton isolation; of these, single peloton isolation (i.e., the isolation of pelotons from host cells by micromanipulation) is considered as the most reliable and accurate method (Zettler et al., 2005; Batty et al., 2006; Zi et al., 2014; Zettler and Corey, 2018).

Molecular identification methods mainly consist of traditional research approaches as well as sequencing *via* high-throughput platforms. Traditional methods include DNA microarrays, clone libraries, denaturing gradient gel electrophoresis, fluorescence *in situ* hybridization, and gene chips (Dearnaley, 2007; Tedersoo and Nilsson, 2016). Considering the shortcomings of these traditional methods, such as low throughput, tedious operation framework, and low accuracy, and the development and popularity of MiSeq PE300 and HiSeq PE250 platforms, the molecular identification of orchid-associated fungi has been improved, and these techniques have replaced the fastidious cloning techniques (Julou et al., 2005). HTS technologies have several advantages such as high throughput, low cost, objective reduction of microbial community structure, and trace detection of fungi (Cruz et al., 2014; Tedersoo and Nilsson, 2016; Tedersoo et al., 2018; Nilsson et al., 2019). However, they also allow the amplification of contaminants and non-OM fungi. In addition, the recent shotgun metagenomic technology using an Illumina NovaSeq/Hiseq sequencer can acquire functional gene information from all the microorganisms in a community through genomic DNA analysis (Bahram et al., 2018; Fadiji and Babalola, 2020). Together with the increasing availability of orchid and reference OM fungal genomes (Kohler et al., 2015; Zhang et al., 2016, 2017), this technology promises extraordinary progress in the study of OM.

Although isolation methods can neither acquire the uncultivable fungi, comprising a large proportion, nor determine all the taxonomic data, this method remains crucial for experiments and determining fungal functions in the symbiosis (Hyde and Soytong, 2008; Sathiyadash et al., 2012; Oliveira et al., 2014; Salazar-Cerezo et al., 2018; Raza et al., 2019). In order to effectively utilize fungi for the orchid protection and research on orchid physiology *ex situ*, it is essential to use the various types of culture media in order to isolate all the fungi that are potentially relevant for orchid growth. For this, we support single peloton isolation as the most reliable method if combined with high-efficiency molecular identification, especially if carried out at different developmental stages and in various habitats.

## Primer Selection in HTS

In fungal research, various PCR primers targeting ribosomal RNA (rRNA) locus, mainly the internal transcribed spacer (ITS), were used in the initial OM diversity studies (Selosse et al., 2004; Shefferson et al., 2005; Bonnardeaux et al., 2007). However, an accelerated rDNA sequence complicates the amplification of one of the most common OMF taxon (Taylor et al., 2002; Binder et al., 2005), the family Tulasnellaceae (see below), driving the development and optimization of OM-specific PCR primers.

Taylor and McCormick (2008) first developed specific primers ITS1-OF and ITS4-OF for studying OM diversity by amplifying the full ITS based on the sequences of Basidiomycete fungi including *Tulasnella* species. This set has been widely used for the identification of OM *via* cloning and sequencing (Xing et al., 2015, 2017, 2019; Jacquemyn et al., 2016a; Kaur et al., 2019; Rammitsu et al., 2019). The ITS4Tul primer designed by Taylor (1997) is also widely used to look for *Tulasnella* diversity along with ITS1 or ITS5 primers (Bidartondo et al., 2003; Abadie et al., 2006; Taylor and McCormick, 2008; McCormick et al., 2012, 2016). Later, an *in silico* analysis suggested that ITS3/ITS4OF and ITS86F/ITS4 primers, targeting the ITS2 sub-region of ITS, were the most ideal for orchid root samples (Waud et al., 2014), even if they not always perform well on soil samples. Moreover, because ITS2 can produce more operational taxonomic units (OTUs) and higher phylogenetic richness than the other sub-region ITS1, many researchers prefer it for fungal identification through HTS platforms (Mello et al., 2011; Tedersoo and Lindahl, 2016; Nilsson et al., 2019). In the past half-decade ITS3/ITS4OF were frequently used for identifying mycorrhizal fungal communities in orchid roots of terrestrial orchids and the surrounding soils (Jacquemyn et al., 2015a, 2017b; Esposito et al., 2016; Duffy et al., 2019). Additionally, ITS86F/ITS4 is still used for the detection of mycorrhizal partners of epiphytic orchids (Cevallos et al., 2017, 2018a; Herrera et al., 2019b; Izuddin et al., 2019; Jacquemyn et al., 2021).

Notably, ITS4OF exhibits four mismatches with 64% of Tulasnellaceae, and multiple mismatches in other assemblages of Basidiomycota and Ascomycota. Similarly, considering that ITS86F has five mismatches in 83% of Tulasnellaceae, Oja et al. (2015) tagged the modified primers ITS1ngs, ITS1Fngs and ITS4ngs, and developed the ITS4Tul2 primer for the full length of ITS. Their integrated utilization matched most of the known mycorrhizal assemblages of orchids (incl. 97% of Tulasnellaceae). Recently, a newly developed primer 5.8S-OF combined with two different reverse primers (ITS4OF and ITS4Tul) revealed good success on OMF (Vogt-Schilb et al., 2020).

Tulasnellaceae (from the order Cantharellales) are the key mycorrhizal symbionts of orchids, mainly its clades A and B. Compared to clade A, clade B is well differentiated and hardly amplified by general primers or even by Tulasnellaceae-specific primers (Girlanda et al., 2011; Lindahl et al., 2013). According to User-friendly Nordic ITS Ectomycorrhiza (UNITE) database dedicated to molecular identification of fungi,^1^ approximately 3/4 of Tulasnellaceae sequences belong to clade A; however, considering strong primer and sampling biases (mainly in the Northern Hemisphere), Tulasnellaceae clade B could be underrepresented, in spite of being equally common (Oja et al., 2015). Therefore, future studies focusing on the diversity of OM should meticulously choose primers and evaluate their potential biases.

One may be aware that no primer set is perfect and consider using several sets available at the time of designing the study. Based on this and a series of works from our research team (unpublished data), it is highly recommended to sue multiple pairs of primers with low amplification overlap. They can be amalgamated and combined with a nested PCR amplification method to identify the maximum number of orchid mycorrhizal partners (Oja et al., 2015; Voyron et al., 2017; Vogt-Schilb et al., 2020). The use of three optimized primer pairs, ITS1ngs-ITS4ngs, ITS1Fngs-ITS4ngs, and ITS1-ITS4Tul2, were recommended for 454 pyrosequencing. For amplicon sequencing using MiSeq PE300 and HiSeq PE250, two primer pairs (Supplementary Figure S1), namely, ITS1F-ITS4 and ITS1-ITS4Tul can be recommended for the first round of amplification (Gardes and Bruns, 1993; Bidartondo et al., 2003). PCR products can be further subjected to nested PCR amplification using ITS86F-ITS4 and ITS86F-ITS4Tul primers, which capture a large diversity of mycorrhizal fungi associated with 72 varieties of tropical epiphytic orchids grown in the wild (Li et al., unpublished). For the third-generation PacBio Sequel sequencing, optimized ITS1ngs-TW14ngs, ITS1Fngs-TW14ngs, and ITS1-ITS4Tul2 primers can be recommended (see Supplementary Table S1 for the above-mentioned primer sequences). In the future, the shotgun sequencing of roots, which provide no PCR amplification, while a fungus can be observed, may allow to unravel clades that escape all primers available, if any.

## Fungal Diversity and Specificity in Orchids

### Orchid-Fungal Diversity

Orchids are often associated with phylogenetically and ecologically diverse fungi. Basidiomycota and Ascomycota, with very few Chytridiomycota, Glomeromycota, Zygomycota, or Mucoromycota, are widely distributed in the aerial roots of epiphytic orchids and in the underground roots or rhizomes of terrestrial and lithophytic orchids (Martos et al., 2012; Waud et al., 2014; Cevallos et al., 2018a; Egidi et al., 2018; Novotná et al., 2018; Qin et al., 2019). Fungi in tissues can be divided into true OMF and orchid non-mycorrhizal fungi (ONF) based on the structures formed during in orchids. Coiled pelotons in root cortical cells are characteristic for OMF (Rasmussen, 1995; Suárez et al., 2009; Dearnaley et al., 2016); ONF are endophytic fungi that colonize roots or other tissues at a certain period during the life span of orchids but possess no peloton-like structures and cause no obvious pathogenic symptoms in host orchids (Rasmussen, 1995; Bayman and Otero, 2006; Selosse et al., 2018; Sisti et al., 2019). Most ONF in root tissues of a given orchid show no noticeable phylogenetic relationship with known OMF (Stark et al., 2009), even if some OMF may be ancient ONF that evolved mycorrhizal abilities (Jacquemyn et al., 2017b; Selosse et al., 2018).

### Orchid Mycorrhizal Fungal Diversity

The most common OMF, belonging to Basidiomycota, are traditionally grouped under the name rhizoctonias, including Tulasnellaceae and Ceratobasidiaceae (belonging to Cantharellales) as well as Serendipitaceae (previously known as Sebacinales clade B). Most of these three fungal taxa have ecological niches ranging (and mixing in many cases) saprotrophy, i.e., exploiting decaying matter, and endophytism in non-orchid plants (Dearnaley et al., 2012; Selosse and Martos, 2014; Jacquemyn et al., 2017a; McCormick et al., 2018; Selosse et al., 2018). This clearly applies for Serendipitaceae at least: the famous endophytic model *Serendipita* (= *Piriformospora*) *indica* is orchid mycorrhizal in Brazilian orchids (Oliveira et al., 2014). In addition, Thelephoraceae fungi are also commonly found in some orchids, such as *Cephalanthera longibracteata* and *Liparis loeselii* (Bidartondo and Read, 2008; Jacquemyn et al., 2015a; Waud et al., 2017; Herrera et al., 2019a). Further, Selosse et al. (2004) observed Ascomycota from the genus *Tuber*, based on molecular identification, transmission electron microscope, and immunolabeling in *Epipactis microphylla*. Ascomycota were sporadically reported, e.g., as rare OMF in MX orchids, such as *Limodorum abortivum* and *Epipactis helleborine* (Girlanda et al., 2006; May et al., 2020; Xing et al., 2020), and in South-African orchids (Waterman et al., 2011).

Some AT orchids are associated with Atractiellales (Pucciniomycotina; Kottke et al., 2010; Cevallos et al., 2018b; Qin et al., 2019; Xing et al., 2019) and saprotrophic fungi from Mycenaceae or ECM fungi from Russulaceae, *Peziza*, and *Inocybe* (Waterman et al., 2011; Zhang et al., 2012; Esposito et al., 2016; Waud et al., 2017; Xing et al., 2020). Additionally, a few photosynthetic orchids associate with saprotrophic Auriculariales, Psathyrellaceae, *Tricharina*, *Clavulina*, *Armillaria*, *Marasmius*, and *Scleroderma* fungi belonging to the common ECM fungal taxa (Waterman et al., 2011; Yagame et al., 2013; Jacquemyn et al., 2016b; González-Chávez et al., 2018; Qin et al., 2019; May et al., 2020; Salazar et al., 2020). The presence of such fungi, likely in minor amounts, was greatly enhanced by the use of HTS and the reporting of all the diversity found, without *a priori* screening (Selosse et al., 2010). Although some MX orchids preserve their autotrophic ability, as shown by intact photosynthetic genes in plastid genomes (Lallemand et al., 2019), they possess few or no rhizoctonias in their roots, whereas ECM fungi are dominant as far as we know (Paduano et al., 2011; Merckx, 2013; Gebauer et al., 2016; Schiebold et al., 2018). MH orchids associate with ECM fungi or non-rhizoctonia saprotrophic fungi, including wood or litter decomposers (Martos et al., 2009; Hynson et al., 2013; Lee et al., 2015; Kinoshita et al., 2016; Ogura-Tsujita et al., 2018). However, a few MH and MX orchids may superficially look like associated with rhizoctonias; for example, the MH *Rhizanthella gardneri* (Bougoure et al., 2009) or the MX *Platanthera minor* (Yagame et al., 2012) with *Ceratobasidium* spp., but which belong to a sub-clade forming ECM.

In all, OMF have been reported from at least 17 families of Basidiomycetes and five families/genera of Ascomycetes (Dearnaley, 2007; Waterman et al., 2011; Dearnaley et al., 2012). Notably, dark septate endophytes with melanized hyphae, mainly include the members of Helotiales with variable impact on roots (Newsham, 2011), have been observed in some AT and MX orchids, adding to the fungal diversity in orchid (Calvert, 2017; Schiebold et al., 2018).

During the orchid life cycle, OMF communities can change, although general trends are established from a limited number of species. Very often (but not always), OMF diversity tends to decline from the seed stage to the seedling stage and to often increase again in the adult stage in an hourglass-like pattern. In terrestrial orchids, OMF from protocorms or seedlings are conventionally subsets of those found in early germinating seedlings and adult plants (Bidartondo and Read, 2008; Jacquemyn et al., 2011a; Waud et al., 2017). This has been observed for culturable *Tulasnella* species (Meng et al., 2019a). In addition, recent investigations indicate continuous OMF community dynamics at the different stages of orchid life cycle (Cevallos et al., 2018b). To summarize, orchids are subject to internal and external natural conditions, potentially promoting symbiotic benefits (McCormick et al., 2016, 2018).

### Orchid Non-mycorrhizal Fungal Diversity

The ecological adaptability of ONF facilitates their wide distribution, covering over 110 genera, of which 76 genera belong to Ascomycetes with much higher diversity and occurrence frequency than those of OMF (Sudheep and Sridhar, 2012; Ma et al., 2015). The cultivability of most of them makes their isolation relatively easy. Xylariales (e.g., *Xylaria* spp. and *Hypoxylon* spp.) and Helotiales (e.g., Helotiaceae and Hyaloscyphaceae) are the main ONF associated with tropical and temperate orchids, respectively; Chaetothyriales, Hypocreales, Helotiales, and Capnodiales are also frequent in tropical epiphytic orchids (Bayman et al., 1997; Dearnaley et al., 2012; Oliveira et al., 2014; Govinda Rajulu et al., 2016; Jacquemyn et al., 2016a; Beltrán-Nambo et al., 2018; Novotná et al., 2018). In addition, *Colletotrichum*, *Fusarium*, and *Trichoderma* fungi are generally found in the roots of various orchids from tropical and temperate zones (Martos et al., 2012; Tao et al., 2013; Sufaati et al., 2016; Salazar-Cerezo et al., 2018; Sisti et al., 2019; Sarsaiya et al., 2020).

These taxa are well-known endophytes in many plants (Rodriguez et al., 2009; Selosse et al., 2018) and entail no known disease symptoms. In contrast, a very small number of potential plant pathogens have been proven to support the growth and development of orchids to some extent. For example, *Fusarium* fungi are known to promote an early seed germination of some *Cypripedium* and *Platanthera* spp. (Vujanovic et al., 2000; Bayman and Otero, 2006), and *Fusarium* have been suggested to be OMF in a few orchids (Jiang et al., 2019; Sisti et al., 2019). Interestingly, *Colletotrichum* enhance the growth of adult individuals belonging to *Dendrobium* spp., despite its high pathogenicity on seedlings (Shah et al., 2019; Sarsaiya et al., 2020).

Currently, *in vitro* investigations on the functions of ONF during the life cycle of orchids are limited, even though at least 65 ONF genera have been successfully isolated and cultured (Novotná et al., 2018; Meng et al., 2019a,b,c; Sarsaiya et al., 2019; Shah et al., 2019; Bell et al., 2020). It is crucial to further investigate the ONF that frequently occurs in orchids roots, in order to determine their potential physiological and ecological advantages. Active compounds produced by some ONF may prove beneficial for orchids by improving their resistance to abiotic stresses, thereby promoting their adaptability to different environmental conditions (Ma et al., 2015) or by protecting against pathogens and herbivores. Some ONF may even decompose local substrates and provide some nutrients to the orchids (Tedersoo et al., 2009; Waterman et al., 2011; Herrera et al., 2019a). Considering the potential significance of ONF, it is crucial to analyze the balance between OMF and ONF (including potential pathogens) in orchid ecology (Nilsson et al., 2019).

### Orchid Mycorrhizal Fungal Specificity

Orchids interact with a more limited set of mycorrhizal fungi as compared to other mycorrhizal plants, with a relatively higher degree of specificity for OM than ECM, AM, and even ErM fungi (Dearnaley et al., 2012; van der Heijden et al., 2015; Suárez and Kottke, 2016; Põlme et al., 2018). Mycorrhizal specificity, one of the core issues in OM research, ranges from low to high and can be quantified as the phylogenetic width of the range of associated mycorrhizal fungi (i.e., the ancientness of the last common ancestor). Moreover, despite some phylogenetic conservatism in mycorrhizal partners (Martos et al., 2012) and specificity in orchids (Jacquemyn et al., 2011b), specificity level is a labile evolutionary trait (Irwin et al., 2007; Shefferson et al., 2007, 2010). According to the degree of specialization and the ecological opportunities for interactions and following Shefferson et al. (2019), specificity can be subdivided into assemblage specialization (combined with specific orchid hosts; the interactions are less affected by the environment), apparent generalism (combined with a few orchid hosts; there is a certain correlation between interactions and the environment), and true generalism (combined with multiple orchid hosts; the interactions are greatly affected by the environment).

The OM specificity may be affected by environmental factors, climate changes, extreme host selections, evolutionary history, accompanying plant species, biocompatibility, biogeographic range, and density of OMF in the soil or, for epiphytes, phorophytes (Jacquemyn et al., 2011b, 2017a; McCormick et al., 2012, 2018; Pandey et al., 2013; Waud et al., 2016a; Shefferson et al., 2019; Xing et al., 2019), thus showing strong and complex variations. For example, *Neottia* and *Caladenia* prefer symbiosis with sebacinales fungi (Těšitelová et al., 2015; Phillips et al., 2016; Reiter et al., 2020); the rare terrestrial orchid *Caladenia huegelii* specifically associates with *Serendipita (= Sebacina) vermifera* (Swarts et al., 2010); *Cypripedium*, *Ophrys*, and *Chiloglottis* prefer Tulasnellaceae (Shefferson et al., 2005, 2019; Roche et al., 2010; Schatz et al., 2010); *Dendrobium nobile* and *Liparis japonica* have high specificity for Tulasnellaceae (Ding et al., 2014; Xing et al., 2017); *Pterostylis nutans* and *Sarcochilus weinthalii* are only symbiotic with *Ceratobasidium* fungi (Irwin et al., 2007; Graham and Dearnaley, 2012); *Platanthera leucophaea* tends to be associated with *Ceratobasidium* fungi over a 10-year period (Thixton et al., 2020); *Corallorhiza trifida* shows high specificity for Thelephoraceae in different countries and varied habitats (McKendrick et al., 2000; Zimmer et al., 2008).

Jacquemyn et al. (2010) asserted the possibility of promoting widespread associations between orchids and available OMF in an environment devoid of water and nutrients. However, despite the diminished availability of groundwater and nutrients, the associated Tulasnellaceae showed an increasing trend of specificity from terrestrial to epiphytic and lithophytic orchids (Xing et al., 2019). Moreover, OMF of lithophytic orchids are mostly Tulasnellaceae. The specificity may also differ between biogeographic regions. Mainland Australia appears to have a relatively higher incidence of mycorrhizal specificity than, e.g., South Africa, Eurasia, and North America (Roche et al., 2010; Phillips et al., 2011, 2016; Davis et al., 2015; Jacquemyn et al., 2017a; Reiter et al., 2020). For instance, the genus *Corycium* of subtribe *Coryciinae* is almost exclusively symbiotic with *Peziza*, and all orchids in *Disperis* only associate with Ceratobasidiaceae (Waterman et al., 2011; Jacquemyn et al., 2017a). Furthermore, MX orchid mycorrhizae often have lower specificity than AT and MH and culmulatively associate with various ECM fungi or rhizoctonias (Abadie et al., 2006). However, there are a few specific MX orchids, such as *P. minor* and *L. abortivum* that specifically associate with ECM *Ceratobasidium* fungi and *Russula delica*, respectively (Girlanda et al., 2006; Yagame et al., 2012). In a *Pinus thunbergii* plantation, the MX *E. helleborine* displayed abundant *Wilcoxina* fungi, but whether this is an OMF remains unclear (Suetsugu et al., 2017), while this species is normally poorly specific (May et al., 2020).

A few studies address the specificity of OM at different stages of orchid life cycle. Bidartondo and Read (2008) detected fungus-specific bottleneck at the seedling stage of *Cephalanthera* spp. Těšitelová et al. (2012, 2015) studied the OMF assemblages of *Neottia* and *Epipactis* species in protocorms and adult, indicating that differences in OMF diversity were scarce (as for the single *Epipactis* species studied in Bidartondo and Read, 2008). Recently, OMF identification in a large number of samples of the Japanese epiphytic *Taeniophyllum glandulosum* revealed specific link to Ceratobasidiaceae throughout the entire life cycle and whatever the phorophyte (Rammitsu et al., 2019). *Ceratobasidium* spp. highly specifically associate with two rare and endangered *Bipinnula* orchids at the adult stage and effectively promote seed germination (Claro et al., 2020). However, the specificity of OMF sometimes varies at different developmental stages. For example, *Gastrodia elata* relies on fungi, such as *Mycena osmundicola*, during seed germination and protocorm development, while adults associate with *Armillaria mellea*, despite it inhibits the germination seeds to a certain extent (Ran and Xu, 1988; Xu and Guo, 1989). The invasive *Oeceoclades maculata* associates with multiple saprotrophic fungi and rhizoctonias at the adult stage; however, during *in vitro* germination, seeds retain high specificity for *Psathyrella* spp. (Bayman et al., 2016).

High OMF specificity is often observed in orchids with habitats specialized or those distributed in the southern hemisphere (Davis et al., 2015). On the one hand, this may be attributed to an extreme selection of host orchids during the recent historical adaptation; while on the other hand, it may be a result of a low local OMF diversity (Illyés et al., 2009; Swarts et al., 2010; Waud et al., 2017). Mycorrhizal specificity does not limit the distribution range or rarity of at least some orchids (Shefferson et al., 2007; Phillips et al., 2011, 2016; Bailarote et al., 2012; Pandey et al., 2013; Waud et al., 2017), since specificity for a widespread fungus is not limitative. Indeed, OMF associated with orchids exhibiting high mycorrhizal specificity tend to have a wide distribution (Jacquemyn et al., 2011b; Swift et al., 2019; Thixton et al., 2020). Even OMF promoting the germination of narrowly distributed orchids may display a wider distribution (Oktalira et al., 2019).

The adaptive significance of OM specificity relies on three points. First, specificity increases the germination rate of seeds and thus improves fitness by survival. Second, it may enhance the efficiency of nutrient exchange between orchids and OMF, thereby promoting the efficiency of bidirectional carbon flow in OM symbiosis, or even, facilitating the absorption of carbon by MH and MX orchids (Bonnardeaux et al., 2007; Suetsugu et al., 2017); such exchange improves fitness by seed set (Roy et al., 2013). Conversely, more effective nutrient exchange is hypothesized one of the driving factors for OM specificity (Selosse et al., 2002; McCormick et al., 2006; Nurfadilah et al., 2013). Third, specificity facilitates the formation of patchy distribution of orchids under natural conditions, and this affects gene flow, population dynamics, and pollination systems (Tremblay et al., 2005).

## Difference and Adaptability of OMF in Different Habitats

**Figure 1:**
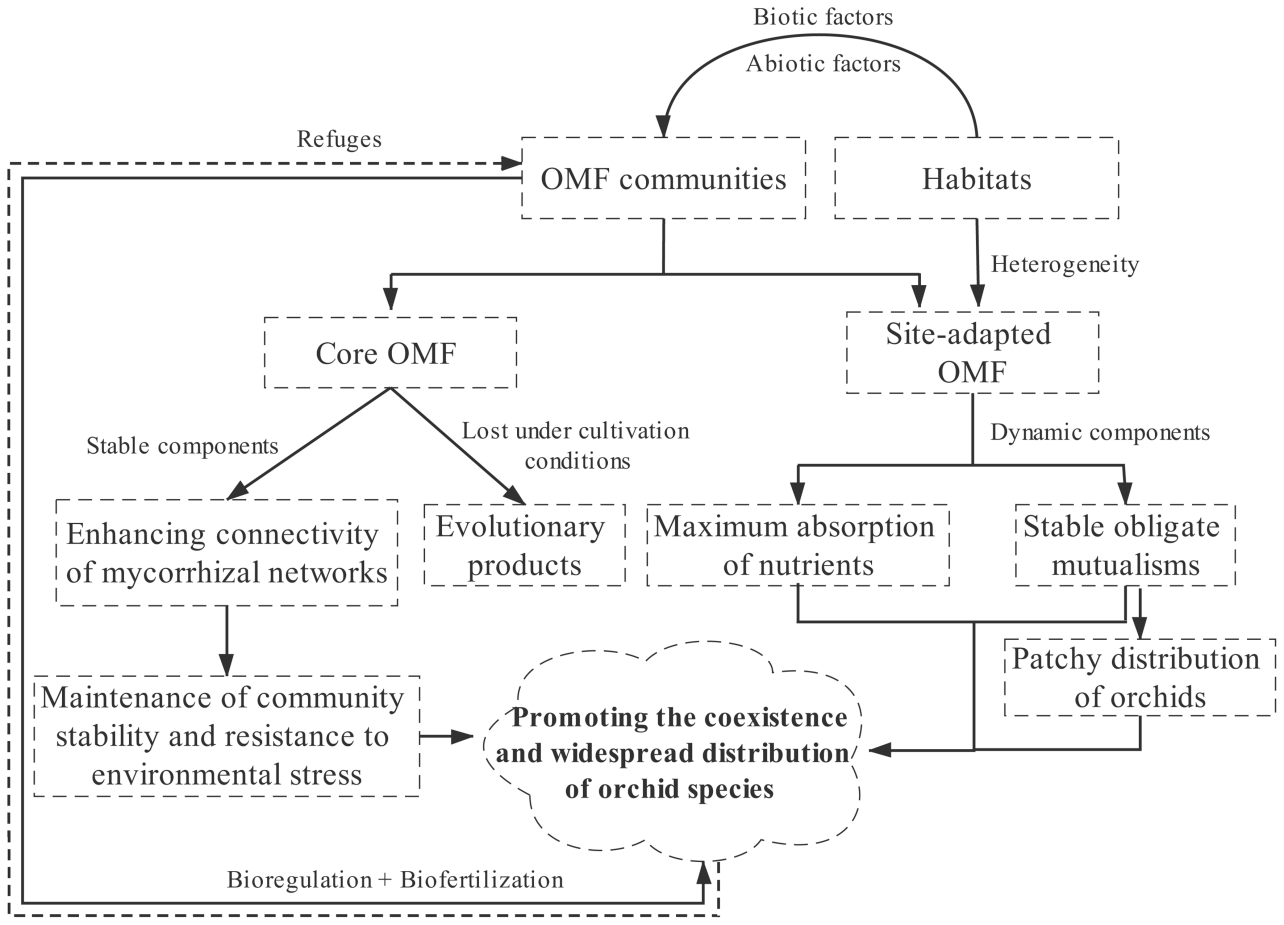
A framework depicting how habitats indirectly affect the coexistence and widespread distribution of orchid species by affecting OMF communities. OMF communities shaped by different habitats affect the growth and development of orchids (bioregulation) by promoting nutrient absorption (biofertilization). The dotted arrow indicates that the current understanding of the impact of orchids on OMF life cycle is very limited.

Habitats, including macro- and micro-habitats, indirectly affect the coexistence and widespread distribution of orchid species by affecting the composition, structure, and richness of OMF communities (Figure 1). The community composition of OMF associated with the same orchid species distributed in different habitats, different orchids co-occurring in the same habitat, or different individuals of most orchid species in the same habitat can differ to some extent (Martos et al., 2012; Jacquemyn et al., 2014, 2015a, 2016a, 2021; Waud et al., 2017; Pecoraro et al., 2018; Duffy et al., 2019).

From a macrohabitat perspective, most orchids distributed in the Mediterranean shrublands, temperate deciduous forests, boreal forests, and tropical forests are associated with a large number of mycorrhizal partners, with the most prominent OMF families being Tulasnellaceae and Ceratobasidiaceae, ECM basidiomycetes, Sebacinaceae and Thelephoraceae, and rhizoctonias (*Tulasnella* as the most common taxon; Jacquemyn et al., 2017a). Concurrently, habitat heterogeneity or local environmental conditions also substantially affect the OMF communities of different populations or at different sites of the same species of orchids. For example, for nine *Neottia ovata* populations of different sizes, the orchid individuals in the most central part exhibit significantly different OMF communities (Jacquemyn et al., 2015b). Oja et al. (2015) corroborated that *N. ovata* in grasslands and forests have different OMF compositions (see also Těšitelová et al., 2015). Of note, greater habitat differentiation may lead to more significant mycorrhizal association variation among *Dactylorhiza* species inhabiting different sites than among *Orchis* species (Jacquemyn et al., 2011b, 2016b). Considering the continent-wide scale, strong turnovers are witnessed in the OMF communities associated with *Gymnadenia conopsea* and *E. helleborine* from Europe to China (Xing et al., 2020), although the genetics of the plant itself can somewhat change over such a distance. Furthermore, some AT orchids distributed in the Mediterranean grasslands, Australia, and tropical montane rainforests also display significant changes the OMF in different habitats or populations (Ramsay et al., 1987; Pandey et al., 2013; Jacquemyn et al., 2014, 2016a; Cevallos et al., 2017; Waud et al., 2017; Herrera et al., 2018; Duffy et al., 2019). These reports indicate that the availability of OMF does not restrict the distribution of orchids, but that their occurrence is bounded by specific ecological habitats.

From a microhabitat perspective, sympatric orchids are usually associated with different OMF (Martos et al., 2012; Jacquemyn et al., 2015a, 2016a, 2021; Cevallos et al., 2017; Qin et al., 2019). If two or more species are equally dependent on the same available resources, theoretical models predict that they cannot coexist (niche exclusion); indeed, at small-scale, associating with different mycorrhizal fungi facilitates rational division of the niche space resources among cohabiting species by reducing competition and avoiding indirect exploitation due to asymmetrical benefits from shared fungi (Tilman, 1982; Selosse et al., 2004; McCormick and Jacquemyn, 2014; Gerz et al., 2018). Simultaneous association with multiple OMF taxa may maximize the absorption of nutrients as well as stabilize obligate mutualisms with respect to robustness. Notably, the OMF composition and community structures of *Paphiopedilum dianthum* and *P. hirsutissimum* that often coexist (i.e., almost same microhabitats) on limestone vary significantly (Li et al., unpublished); hence, the co-occurring orchid species with different OMF compositions may also result from a strong OMF selection by the host orchids (Jacquemyn et al., 2015a; Xing et al., 2019).

However, some reports suggest that sympatric orchids, conspecific orchids in different populations or sites, or even epiphytic and terrestrial orchids in different habitats share some generalized OMF. These OMF may have stronger ecological adaptability and are commonly found in orchid mycorrhizal networks, which may maintain community stability under environmental stresses or variations (Kottke et al., 2013; Pandey et al., 2013; Suárez et al., 2016; Pecoraro et al., 2017; Xing et al., 2017; Herrera et al., 2018, 2019b). To better define these specific fungal guilds, Cevallos et al. (2017) proposed the “keystone species” theory based on their study of OMF communities associated with epiphytic orchids: the OMF community of a given orchid species in different sites contains a core (permanent components or keystone species), and site-adapted mycorrhizal fungi (dynamic components). This has also been observed in the OMF communities of seedlings transplanted at different elevation gradients (Cevallos et al., 2018b; Herrera et al., 2019b). Interestingly, studies have shown that some orchids either have completely different OMF or share few OMF under natural habitats and cultivation conditions (Downing et al., 2017; Qin et al., 2019). Therefore, the selection among habitats, host orchids, and OMF is more complicated than previously perceived. Comparative studies at larger scales will shed light on rules followed by these symbiont assemblies.

Similar to all soil fungi, AM, ECM, pathogens, and saprotrophic fungi, the composition and richness of OMF communities also differ according to various ecological factors, such as soil physical and chemical properties (e.g., P abundance), tree richness, altitude, mean annual precipitation, and mean annual temperature (Geml et al., 2014; Barnes et al., 2016; Geml, 2017; Hu et al., 2019; Mujica et al., 2020). Elucidating the interplay between biotic and abiotic factors in shaping OMF communities is vital for conservation practices such as introduction and artificially assisted colonization (the transfer of individuals from the current natural range to potential new habitats to adapt to climate change).

In terms of biotic factors, changes in habitat vegetation influence the OMF communities associated with orchid roots (Pandey et al., 2013; Herrera et al., 2018), by changing soil or even plants in which rhizoctonias are endophytes. Especially, some terrestrial MX or MH, if not AT, orchids often tend to share fungi with surrounding trees (Bidartondo and Read, 2008; McCormick et al., 2009; Jacquemyn et al., 2016a). Moreover, orchid species living on different phorophytes or different epiphytic niches on the same phorophyte tend to associate with different OMF (Wang et al., 2017; Rasmussen and Rasmussen, 2018), although broader validation of this expected.

With respect to abiotic factors, several studies suggest that many soil characteristics (e.g., soil water content, pH, organic matter content, nutrition level, trace elements, and textural components) explain the distribution of OMF in habitat patches (Jacquemyn et al., 2015b; Esposito et al., 2016; Waud et al., 2017; Kaur et al., 2019; Vogt-Schilb et al., 2020). Depending on the model, the OMF communities seem to be significantly related to the hydrolyzable and extractable nitrogen in the soil (Han et al., 2016; Duffy et al., 2019) and to the P availability (Mujica et al., 2020), but a general view is pending, beyond existing case studies. Factors, such as rainfall and humus type, predict and affect the presence and richness of OMF-associated epiphytic orchids (Izuddin et al., 2019). Interestingly, although epiphytic orchids and terrestrial orchids exhibit different responses to light at the early growth, OMF have parallel ecological importance in overcoming the photoinhibition during germination and early growth (Alghamdi, 2017).

Considering the effect of latitude on the species and functional group richness of OMF in orchids, diversity decreases with increasing latitude; however, whether the orchid diversity follows similar trends remains unclear (Jacquemyn et al., 2017a; Duffy et al., 2019). The diversity of orchids has been proven to initially increase and then decrease with increasing altitude, with a peak in the mid-altitude area (approximately 1,000–1,600 m; Küper et al., 2004; Cardelús et al., 2006; Acharya et al., 2011). At present, no reports suggest changes in OMF communities under an altitudinal gradient. Studies on high-altitude tropical southern Ecuador montane rainforests reported that some epiphytic orchids have higher richness or abundance of OMF at relatively high altitudes (Cevallos et al., 2018a; Herrera et al., 2019b). However, Cevallos et al. (2018a) insisted that high-altitude areas (3000–3,500 m) showed no significant difference in fungal communities among different orchid species or sites, while in low-altitude areas (2,050–2,800 m), host orchids and sites were the two major drivers of OMF communities’ composition. Cevallos et al. (2018b) suggest that OMF display rich diversity in mid-altitude areas (approximately 2,050 m), but this is limited to a narrow altitude range (1,850–2,100 m). Therefore, whether the OMF composition and community structure are opportunistic associations (Dearnaley et al., 2012) or exhibit certain trends under large altitudinal gradients is still under scrutiny.

At the same latitude, orchids in areas with higher temperatures and fewer seasonal changes exhibit higher OMF diversity. Environmental conditions, such as high temperature or high humidity, may increase the activity of some saprotrophic fungi, enabling them to obtain more carbon so as to support the growth of MH orchids (Martos et al., 2009; Shefferson et al., 2019). Recently, it was proposed that the colonization of some dominant OMF in orchid roots is significantly affected by rainfall (McCormick et al., 2006; Kartzinel et al., 2013; Jasinge et al., 2018). Among these, Tulasnellaceae increase with increasing rainfall, indicating their hydrophilic nature. Similarly, Ceratobasidiaceae, a dominant assemblage in dry habitats, displays a higher resistance to drought. Thus, climate may also be a major driver for the turnover of orchids and OMF at different sites (Kottke et al., 2013).

In conclusion, climatic changes may strengthen the filtering effect of environmental factors on OMF associated with orchids by perturbing biological interactions. Furthermore, in addition to ecological factors, the phylogeny, ploidy levels, and genome composition of host orchids as well as the sampling effort itself influences the OMF communities (Shefferson et al., 2007, 2019; Jacquemyn et al., 2011b, 2016b; Těšitelová et al., 2013). Therefore, OMF diversity is expected to intertwine multiple factors.

## Comparison of OMF in Orchid Roots and Soil

Despite extensive research on the diversity of OMF colonizing orchid roots, their spatial distribution and abundance in soil has received more limited attention. Several reports suggest that OMF either exist in saprophytic form in the soil or form ECM or endophytic colonization on adjacent plants (Girlanda et al., 2011; Dearnaley et al., 2012; van der Heijden et al., 2015; Oberwinkler et al., 2017); however, the distribution of OMF in soil remains unclear (Voyron et al., 2017). Analyzing and understanding this is vital for restoring the populations of endangered orchids and for artificially-assisted colonization.

Early seed germination experiments and a few pieces of molecular evidence revealed that most OMF distributed in orchid roots are also widely present in the soil. Moreover, with increase in radial distance between the soil sampling point and the adult orchid, the corresponding OMF richness and abundance often decreased (Table 1). Interestingly, the overall similarity between OMF communities in orchid roots among different populations was higher than that in the soil (Jacquemyn et al., 2015b), whereas many microhabitats near the adult orchids did not support seed germination due to the absence of suitable OMF (Taylor et al., 2010; McCormick et al., 2016; Waud et al., 2016a). These results clearly indicate that OMF are unevenly distributed in the soil and that OMF community display small-scale patchiness in the soil, as other mycorrhizal fungi (Richard et al., 2005; Pickles et al., 2010). This likely contributes to the clumped distribution and patchiness of orchids, as common observed for terrestrial orchids.

**Table 1:** Comparison of OMF in orchid roots and soil.

Orchid species	Number of Orchid species^a^	Methods	PCR primers	Number of PCR primer pairs^b^	OMF in orchid roots		References
					was comparable to that in soil^c^	was sporadic or undetected in soil	
*Caladenia arenicola*	1	Seed germination	Uninvolved	Uninvolved	Yes	No	Batty et al., 2001
*Neottia nidus-avis*	1	Seed germination	Uninvolved	Uninvolved	Yes	No	McKendrick et al., 2002
*Goodyera pubescens*	1	Seed germination	Uninvolved	Uninvolved	Yes	No	Diez, 2007
*Anacamptis morio* *Gymnadenia conopsea* *Orchis mascula*	3	Seed germination	Uninvolved	Uninvolved	Yes	No	Jacquemyn et al., 2012
*Anacamptis morio* *Gymnadenia conopsea* *Orchis mascula*	3	454 pyrosequencing	ITS86F/ITS4 ITS3/ITS4OF	2	Yes	No	Waud et al., 2016a
*Orchis mascula* *Orchis purpurea*	2	Spatial point pattern analysis + qPCR	OTU1f_1g/Tul_r1 OTU2f_2g/Tul_r3	2	Yes	No	Waud et al., 2016b
*Goodyera pubescens* *Liparis liliifolia* *Tipularia discolor*	3	Seed germination + ABI Sequencing + qPCR	GIS-B159 F/R SW-2779-59-1 F/R ITS-Lip1/ITS4-tul Tip14F/Tip14R Tip2_F1/Tip14R ITS5/ITS4-tul	6	Yes	No	McCormick et al., 2016
*Corallorhiza odontorhiza*	1	ABI sequencing + Seed germination	SSU1318-Tom/LSU-Tom2 ITS1-P/ITS4 SSU1318-Tom/ITS-Tom4 SSU1318-Tom/ITS4 ITS1-F/LSU-Tom2	5	Yes	Yes	McCormick et al., 2009
*Cypripedium calceolus* *Neottia ovata* *Orchis militaris*	3	454 pyrosequencing	ITS1ngs/ITS4ngs ITS1Fngs/ITS4ngs ITS1/ITS4-Tul2	3	No	Yes	Oja et al., 2015
*Neottia ovata*	1	454 pyrosequencing	ITS1OF-C/ITS4OF ITS1OF-T/ITS4OF	2	No	Yes	Jacquemyn et al., 2015b
*Paphiopedilum spicerianum*	1	Illumina MiSeq sequencing	ITS3/ITS4OF	1	No	Yes	Han et al., 2016
*Anacamptis morio* *Ophrys sphegodes*	2	Illumina MiSeq sequencing	ITS1F/ITS4 ITS3mod/ITS4	2	No	Yes	Voyron et al., 2017
*Platanthera praeclara*	1	Illumina MiSeq sequencing	ITS1-OF/ITS4-OF ITS1/ITS4-TUL	2	No	Yes	Kaur et al., 2019

a number of orchid species in the first column corresponding to each reference in the last column.

b number of PCR primer pairs in the fourth column corresponding to each reference in the last column.

c distribution and abundance of the potential OMF in soil decreased with an increase in the sampling distance from adult orchids.

Growing evidence proves a higher abundance of OMF in orchid roots than in surrounding soil, thus deserving further investigation. Several studies have stated that the germination rate of seeds remains constant regardless of the presence or absence of adult orchids, whereas the physical and chemical properties of soil have significant effects on seed germination (McCormick and Jacquemyn, 2014; Waud et al., 2017). This suggests that the richness and abundance of OMF are not correlated with the distance from the host orchids, as previously reported. In concert with this, increasing molecular evidence supports the widespread dominance of OMF in orchid roots that either have sporadic occurrence or are undetected in the soil (Egidi et al., 2018), while a few OMF that specifically associate with roots exist with very low relative abundance in orchid-occupied locations (Table 1). Moreover, Voyron et al. (2017) found that OMF displayed significant horizontal spatial autocorrelation in soil, whereas their relative abundance had no significant correlation with the distance from adult orchids. In addition, dominant OMF in the tubers of the chlorophyll-free orchid *Gastrodia flavilabella* were rarely found in the surrounding soil (Liu et al., 2015b).

These reports further support the proposal put forth by Selosse (2014) that the orchid roots could possibly be a protective refuge for some fungi that protects them from external factors (Figure 1), at least in some seasons. Thus, some OMF abundant in orchid roots are absent in the surrounding soil, at least for a part of the year. On one hand, independent or opposed spatiotemporal changes in OMF distribution between orchid roots and soil could explain their absence in the soil at the time of root sampling, although the presence of OMF communities in soil looks relatively stable with almost no turnovers during the entire vegetation growth period (Oja et al., 2015; Voyron et al., 2017). On the other hand, it may be because the OMF belong to short-distance exploration type, i.e., the kind of mycorrhizal fungi that efficiently collect nutrients in a limited volume of soil around the rhizosphere (Peay et al., 2011; Voyron et al., 2017). This was confirmed by a study combining the rhizosphere soil and orchid-occupied bulk soil of the Australian *Diuris fragrantissima*, where a *Tulasnella* fungus was only detected in the rhizosphere soil (Egidi et al., 2018). Additionally, the fungi that take refuge in orchid roots may have distinct ecological characteristics. For instance, *Tulasnella* spp. cannot absorb nitrate in the soil, whereas habitats rich in nitrogen and phosphorus tend to have more abundant *Ceratobasidium* fungi (Nurfadilah et al., 2013; Mujica et al., 2016, 2020; Fochi et al., 2017; Vogt-Schilb et al., 2020).

In summary, discrepancies in the distribution of OMF between adult roots and surrounding soil were revealed by seed germination experiments and barcoding. Several factors may have affected the results in each study, such as the physical and chemical properties of the soil, the difference in amplification ability of the same primer in orchid roots and soil, the quality and volume of samples analyzed for molecular identification, the time and site of experiment, and the specificity of each species. Therefore, seed germination and barcoding should be optimized and combined in the future to explore the correlation between OMF communities in roots and in soil. The current discrepancies regarding this issue also pose a great challenge; specifically, do OMF affect the distribution of orchids or orchids affect the distribution of OMF?

## Spatiotemporal Variation Patterns of OMF

The spatial and temporal linkages between host orchids and their associated OMF communities remain largely unexplored. In the past decade, the combination of *in situ* experiments, isolation and culture *in vitro*, and molecular identification of extensive root samples under natural conditions has led to an understanding of spatiotemporal variations of OMF communities, giving some clues on their control.

### Spatial Variation Patterns

As described in the previous section, there are significant spatial variations in OMF in soil. Similar to other mycorrhizal types and general soil fungal communities at small scales, the distribution of OMF in the soil shows non-random spatial distribution characteristics, and a significant horizontal spatial autocorrelation within 10 m of adult orchids is observed (Waud et al., 2016b; Voyron et al., 2017). In contrast, the detection results of fungi in the soil at a distance of 0–32 m from two host orchids (*Orchis militaris* and *Platanthera chlorantha*) in 21 semi-natural calcareous grasslands suggested that the available evidence for spatial clustering of OMF on a local scale was limited (weak spatial structure), while ONF displayed a more distinct spatial distribution pattern (Oja et al., 2017). The characterization of *Platanthera praeclara*-associated OMF naturally distributed in the tallgrass prairie in North America was studied for many years, and almost all populations and several phenological stages of different years demonstrated high spatiotemporal specificity to a certain Ceratobasidiaceae fungus (Kaur et al., 2019).

Although previous studies provided the evidence that there is no or only minute effect of geographical proximity on the similarity between OMF communities (Jacquemyn et al., 2015b, 2016a; Swift et al., 2019), the recent transcontinental comparisons of OMF associated with two widely distributed Eurasian terrestrial orchids revealed that the OMF community composition of the two orchids has significant turnover in Europe and China, and the similarity between OMF communities decreases significantly with increasing geographical distance (Xing et al., 2020). Significant progress has been made with respect to the spatial distribution of OMF in recent years; however, there are limited reports on the fine spatial distribution of OMF in the different parts of root segments of different ages, different tissues of orchids (i.e., plant niches), and different parts of cortical cells (middle, close to the epidermis, close to the steles, etc.), which should be given more attention in the future.

### Temporal Variation Patterns

Compared to spatial distribution, the contemporary understanding of OMF temporal dynamics is more limited. Orchids are often associated with different OMF at different stages of their life cycle and during their growth and development, and these shifts may mediate their health and ecosystem service functions. Early studies on the temporal variation of orchid-fungal symbionts revealed that seasonal OMF turnover might occur in orchids with annual underground structures (Taylor and Bruns, 1999), which was later confirmed in few tuber-shaped orchids (Huynh et al., 2009; Kohout et al., 2013; Oja et al., 2015). Nevertheless, there are increasing examples of variations in OMF associated with perennial root system orchids over time (Rasmussen and Whigham, 2002; Oja et al., 2015; Jasinge et al., 2018).

A recent study demonstrated that extremely heavy rainfall may drive *Ceratobasidium* sp., which is usually the dominant mycorrhizal fungus colonizer in *Pterostylis revoluta*, to elimination by *T. calospora*; *Ceratobasidium* sp. re-dominates after the magnitude of rainfall returns to a normal level (Jasinge et al., 2018). The fungal community composition and the most abundant fungal genera of the critically endangered *P. spicerianum* tend to vary in rainy and dry seasons, and the abundance of Tulasnellaceae declines significantly during the dry season (Han et al., 2016). Similarly, Kartzinel et al. (2013) found that the reduction in the diversity of Tulasnellaceae associated with a rare tropical epiphytic orchid was related to reduced seasonal precipitation. Furthermore, although the infection rate of *Tulasnella* fungi in the roots and tubers of *Pseudorchis albida* is higher during summer (Kohout et al., 2013), the molecular identification of pelotons isolated manually from *Anacamptis morio* during the complete growing season indicates that *Tulasnella* fungi are more common during winter and autumn, while *Ceratobasidium* and Pezizacean clade tend to dominate during summer and spring, respectively (Ercole et al., 2015). These results provide strong proof that interactions between orchids and OMF are closely related to the season, if not seasonal climatic changes. At a finer time scale, Oja et al. (2015) confirmed the turnover of OMF colonizing the roots of *N. ovata* to be 1 month and that their richness increases at the beginning of the flowering period. Interestingly, the diversity and richness of the detected total fungi tended to show a similar trend, and the flowering period was often accompanied by a significant enrichment of ^15^N (Herrera-Rus et al., 2020; Zeng et al., 2021). One of the explanations for the high diversity of fungal partners at flowering period could be host orchids recruit a large number of fungal assemblages that can supplement the nutrients consumed by blooming by releasing specific signals, and that they may even be crucial to the successful completion of the pollination process. In addition, the lower proportion of OMF in the soil of meadow habitats also demonstrated a slight but significant turnover over time (Oja et al., 2015).

Most of the above-mentioned reports mainly focused on adult orchids, and other developmental stages were only studied by Cevallos et al. (2018b); they combined the seedling trapping experiment and molecular identification to confirm that the composition of the OMF community associated with the seedlings of two epiphytic orchids showed significant temporal variation. In addition, seasonal (or phenological) variation in the diversity of most mycorrhizal types (including OM) is generally only sampled for 1 year at present. Thus, whether these turnovers follow interannual cycles and whether the dynamic fungal assemblages can be attributed to the soil fungal community changes require in-depth explorations at interannual scales across several sampling sites.

### Driving Force for Spatiotemporal Turnovers

Recent studies have proposed that the turnover rate of fungal communities observed in space is relatively smaller than the annual turnover rate; the short-term fluctuations in community abundance are mainly affected by spatial variability, while long-term fluctuations are influenced by the time variability factor (Averill et al., 2019; Ji et al., 2019). The mechanism that drives the spatiotemporal turnovers of OMF is unlikely to be the same; host orchids may select different fungal associates with spatiotemporal variation (Jacquemyn et al., 2015a; Oja et al., 2015; Xing et al., 2019), while different degrees of spatiotemporal dynamic turnovers of the fungal species pool in soil may exist (Ercole et al., 2015). Certain environmental and climate covariates can be used to deduce a few spatiotemporal effects.

Further, the extent to which these spatiotemporal variations depend on the nutrition strategies of host orchids, random dissemination process of OMF, habitat types, seasonality, functional differences within roots, and succession remains to be elucidated. From the perspective of nutrition strategies, the spatiotemporal turnovers of OMF can be attributed to (1) the different nutritional requirement of host orchids at different spatiotemporal points; (2) the differences in the ability of OMF to grow in soil and provide nutrients; and (3) the different adaptability of OMF to environmental conditions (especially harsh environments). Notably, fungal dormancy may play a key role in the spatiotemporal turnover of fungal communities, since dormancy can bypass environmentally imposed choices and allow the consistent presence of genetic variations in the environment, thereby increasing the genetic diversity of fungal communities (Cordovez et al., 2019).

## Future Directions

Fungi are a major pillar of biodiversity in ecosystems, occupying a wide range of niches. Owing to the relatively easy growth of some fungi, fungal diversity and strain collection have always been hot spots in the field of fungal research (Hyde et al., 2019). OM symbiosis is an excellent model for investigating the biological interactions between plants and fungi that would help to solve the fundamental questions on interactions between aboveground and underground pollinators and fungi, respectively (Selosse, 2014; Favre-Godal et al., 2020). Orchids have complex symbiotic relationships with fungi at various stages of their life cycle. Understanding of OMF diversity is just the beginning of a series of interesting scientific explorations.

The emergence of culture methods that differ from traditional technologies, such as genomic information methods based on membrane protein expression and microfluidic technology (Tang, 2020), brings hopes for solving the fungal-specific bottleneck faced by some orchid species that need specific fungal switches to move onto the adult developmental stage after germination. Moreover, considering the continuous development of sequencing technologies, it is expected that the functional diversity of orchid-fungal communities and the complexity of spatiotemporal dynamics will be deciphered in the near future.

The roles played by the deterministic processes based on niche theory and the stochastic processes based on neutral theory in the construction of communities have been well-established in the field of microbial ecology (Dini-Andreote et al., 2015; Xun et al., 2019; Gao et al., 2020a). However, the relative importance of these two processes in shaping OMF communities and the factors controlling them are still undetermined. Nevertheless, based on this review, deterministic processes seem to contribute to the construction of OMF communities because environmental conditions significantly affect OMF diversity and abundance. The construction of any given ecological community involves four main ecoevolutionary processes (Vellend, 2016); in addition to the deterministic selection and the stochastic drift that regulates the relative abundance of species, the diversification that generates genetic variation and the dispersal (the movement of individuals across local communities) are also included. The latter two roles in fungal community assembly and function are frequently overlooked. However, they exhibit crucial functions; for example, priority effects and horizontal gene transfer play key roles in niche preemption/modification and adaptability, respectively (Pinto-Carbó et al., 2016; Toju et al., 2018).

Thus, future research on OMF diversity should primarily focus on how the four main ecological processes interact in the assembly and function of OMF communities. In addition to improve certain shortcomings mentioned in the various sections of this review, considering the overall framework required for further research on the fungal diversity of orchids, we have added several keys or difficult issues that are noteworthy for further explorations in the future:

1. Compared to terrestrial orchids, little is known about the OMF in tropical epiphytic orchids and their distribution on phorophytes. The phorophytes of orchids reportedly cover at least 46 plant families (Rasmussen and Rasmussen, 2018). However, the following two questions remain unanswered: why do orchids have a preference for certain phorophytes? Why can some orchids cohabitate on the same phorophyte but others cannot? Therefore, further investigations and experiments, such as *in situ* seed baiting, the transplantation of seedlings, and the identification of OMF contained in barks or substrates of phorophytes at different sites, are needed to elucidate the interactions among orchids, OMF, and phorophytes.
2. The selection method for OMF-specific primers, in addition to the method combining multiple pairs of complementary primers mentioned in this review, can be further improved by employing epicPCR technology, which can focus on two genes in a genome at the same time (Spencer et al., 2016). Moreover, ONF are frequently detected in different tissues of orchids, but their diversity, community structure, and functions exerted in the different stages of orchid life cycle remain ignored. Considering that OMF and ONF coexist in orchid tissues, there is an urgent need to incorporate the latter in the research goals, as an important turning point in orchid mycology. Moreover, promoting research on the interactions between plant pathogens (e.g., *Alternaria*, *Clonostachys*, *Aspergillus*, *Penicillium*, *Phomopsis*, etc.) and OMF in orchid tissues is strongly recommended.
3. Several recent studies have revealed that the difference in relative abundance generated by amplicon sequencing technology is unable to reflect the difference in true absolute abundance of microorganisms in samples, as it ignores the influences of changes in the overall microbial abundance on hosts (Props et al., 2017; Vandeputte et al., 2017; Guo et al., 2020). Although qPCR can be employed to estimate the absolute abundance of specific strains, some strains require specific qPCR primers that are difficult to evaluate and optimize at an early stage. Hence, qPCR is not suitable for studying complex environmental samples. Therefore, to truly reflect on interactions between orchids and fungi, a sophisticated and innovative absolute quantitative technology that integrates ITS amplicon sequencing, qPCR of total fungi, and qPCR absolute quantification of specific fungal strains is required.
4. The determination of whether some singletons and doubletons obtained *via* HTS are truly rare species remains controversial. However, an in-depth analysis of the fungal metabarcoding data obtained from 16 HTS analyses of representative ribosomal RNA gene regions indicates that less than half of these sequences are artifacts (Brown et al., 2015). Moreover, an increasing number of studies have confirmed that rare microbiological taxa are more active than dominant taxa, which play an over-proportional role in ecosystems multifunctionality (Jousset et al., 2017; Chen et al., 2019; Liang et al., 2020). Hence, extensive care should be taken when disregarding singletons and doubletons. It is recommended that subsequent studies concentrate on changes in orchid-associated fungal diversity after incorporating these low-abundance sequences (at least in a separate analysis) and intensify efforts to explore their functions in the construction and maintenance of orchid communities.
5. In recent years, the determination and analysis of core microbiota in environmental samples have attracted a great deal of attention. Current studies on orchid core fungal taxa mainly use the traditional Venn diagrams, which ignore some ecological characteristics (e.g., the coexistence of members in an ecosystem and some of their functions). To improve the efficiency and accuracy of such predictions, it is recommended to use platforms or software, such as MetaCoMET, COREMIC, BURRITO, and PhyloCore, that consider more critical ecological information (e.g., composition, persistence, connectivity, phylogeny, and functional redundancy) to predict and analyze key species in a given microbial community. It is also necessary to standardize their application for orchid-fungal communities based on the effect of various methods, thus facilitating better comparability of core fungal taxa of different or the same orchid species from different case studies.
6. Clarifying the architecture of the OMF networks formed by co-existing orchids can help to comprehend how these hyper-diverse interacting guilds are maintained and co-evolve in their habitats. This can further reveal the species, lineages, or functional taxa that are significant to ecosystem services.
7. Research on orchid-fungal diversity often involves cryopreservation, especially in case of large-scale sampling to study the mycorrhizal networks. This may result in a decline in the vitality of orchid tissues, affecting the OMF. Therefore, it is highly critical to explore the influence of liquid nitrogen or cryopreservation at −80°C on the composition and structure of OMF communities – a prerequisite for truly reflecting the symbiotic patterns of orchids with OMF in natural habitats.
8. Although it is known that OMFs occur in various habitats, not only the rhizoctonia taxa (e.g., Tulasnellaceae and Ceratobasidiaceae) but also some frequent non-rhizoctonia taxa (e.g., *Mycena*, Thelephoraceae, and Russulaceae) demonstrate a relatively wide distribution in orchids. Further investigations are needed to identify (i) their exact ecology, between pure saprotrophy and pure endophytism in non-orchids and (ii) the geographical hot spots of these OMF. This is crucial for the conservation and regeneration of rare and endangered orchids.
9. Once the diversity of fungi associated with orchids distributed in different habitats is clearly understood, the next step is to evaluate and analyze their functions in these communities and in orchids. There are three methods for exploring the ecological functions of orchid-associated fungi. First, culture methods, such as long-term culture, *in situ* culture, or the dilution of culture medium can be used. In addition to the culture methods, which differ from the traditional technologies mentioned at the beginning of this section, the currently uncultured strains should be isolated to a maximum degree. Then, different isolated fungal sets can be used to evaluate their germination- and growth-promoting effects so as to determine their specific functions at the different stages of orchid life cycle. Second, selective fungicides can be used cautiously to eliminate the key fungi linked with orchid growth and stress resistance, and their respective functions may be preliminarily inferred by observing the growth and reproduction of the host orchids above- and under-ground (Bellino et al., 2014; Jacquemyn and Merckx, 2019). Third, the combination of high-resolution microscopy, metagenomes, whole genomes, or transcriptomes *in* and *ex situ* is expected to reveal the dynamic changes in fungal functions of orchids.
10. The exploration of a mycorrhizal fungal colonization model is highly important in determining the carrying capacity of fungal communities and the function of individual fungi in the construction of these communities. In future research, it is recommended to carry out proactive exploration of OMF colonies in the rhizospheres of orchids through a combination of green fluorescent protein-labeled bioreporter systems, isotope labeling, and HTS. Moreover, how OMF propagate, adapt to the microenvironment of orchid roots, and achieve mutual recognition with the host orchids needs to be elucidated.

To summarize, considering the importance of microbiota in plant growth and health, synthetic communities (SynComs) have increasingly become a hot issue in the study of ecology and evolution of plant microbiomes (Cordovez et al., 2019; Liu et al., 2019). The reconstruction of orchid-fungal communities requires a comprehensive understanding of all the aspects mentioned above, and the design of different fungal assemblages with complementary or synergistic traits is recommended. Simultaneously, the relationship among key fungi, pathogens, and other key microbial assemblages (e.g., bacteria and archaea) that are directly or indirectly associated with the orchid-fungal community should be further studied, and the association pattern of orchid-fungal interaction network should be focused on to predict the fungal SynComs that affect orchid development.

With the joint efforts of the growing number of orchid experts from all over the world, the exploration and understanding of elusive and complex mechanisms underlying orchid-fungal interactions are expected to gradually gain momentum and reveal the much sought-after answers.

## Author Contributions

JG and M-AS designed the outline of the manuscript. TL, JG, M-AS, WY, and SW collected the data and wrote the manuscript. M-AS and JG polished the article. All authors contributed to the article and approved the submitted version.

### Conflict of Interest

The authors declare that the research was conducted in the absence of any commercial or financial relationships that could be construed as a potential conflict of interest.

## Footnotes

^1^https://unite.ut.ee/index.php

## References

[ref1] Abadie J. C. Püttsepp Ü. Gebauer G. Faccio A. Bonfante P. Selosse M.-A. (2006). *Cephalanthera longifolia* (Neottieae, Orchidaceae) is mixotrophic: a comparative study between green and nonphotosynthetic individuals. Can. J. Bot. 84, 1462–1477. 10.1139/b06-101

[ref2] Acharya K. P. Vetaas O. R. Birks H. J. B. (2011). Orchid species richness along Himalayan elevational gradients. J. Biogeogr. 38, 1821–1833. 10.1111/j.1365-2699.2011.02511.x

[ref3] Alghamdi S. A. (2017). Influence of mycorrhizal fungi on seed germination and growth in terrestrial and epiphytic orchids. Saudi J. Biol. Sci. 26, 495–502. 10.1016/j.sjbs.2017.10.021, PMID: 30899164 PMC6408697

[ref4] Averill C. Cates L. A. L. Dietze M. C. Bhatnagar J. M. (2019). Spatial vs. temporal controls over soil fungal community similarity at continental and global scales. ISME J. 13, 2082–2093. 10.1038/s41396-019-0420-1, PMID: 31019271 PMC6776031

[ref5] Bahram M. Hildebrand F. Forslund S. K. Anderson J. L. Soudzilovskaia N. A. Bodegom P. M.. (2018). Structure and function of the global topsoil microbiome. Nature 560, 233–237. 10.1038/s41586-018-0386-6, PMID: 30069051

[ref6] Bailarote B. C. Lievens B. Jacquemyn H. (2012). Does mycorrhizal specificity affect orchid decline and rarity? Am. J. Bot. 99, 1655–1665. 10.3732/ajb.1200117, PMID: 23032816

[ref7] Bajpai A. Rawat S. Johri B. N. (2019). “Fungal diversity: global perspective and ecosystem dynamics,” in Microbial Diversity in Ecosystem Sustainability and Biotechnological Applications. eds. Satyanarayana T. Johri B. N. Das S. K. (Singapore: Springer), 83–113.

[ref8] Barnes C. J. van der Gast C. J. Burns C. A. McNamara N. P. Bending G. D. (2016). Temporally variable geographical distance effects contribute to the assembly of root-associated fungal communities. Front. Microbiol. 7:195. 10.3389/fmicb.2016.00195, PMID: 26941720 PMC4766365

[ref9] Bascompte J. Jordano P. (2007). Plant-animal mutualistic networks: the architecture of biodiversity. Annu. Rev. Ecol. Evol. Syst. 38, 567–593. 10.1146/annurev.ecolsys.38.091206.095818

[ref10] Batty A. L. Brundrett M. C. Dixon K. W. Sivasithamparam K. (2006). *In situ* symbiotic seed germination and propagation of terrestrial orchid seedlings for establishment at field sites. Aust. J. Bot. 54, 375–381. 10.1071/BT04024

[ref11] Batty A. L. Dixon K. W. Brundrett M. Sivasithamparam K. (2001). Constraints to symbiotic germination of terrestrial orchid seed in a mediterranean bushland. New Phytol. 152, 511–520. 10.1046/j.0028-646X.2001.00277.x, PMID: 33862990

[ref12] Bayman P. Lebrón L. L. Tremblay R. L. Lodge D. J. (1997). Variation in endophytic fungi from roots and leaves of *Lepanthes* (Orchidaceae). New Phytol. 135, 143–149. 10.1046/j.1469-8137.1997.00618.x, PMID: 33863156

[ref13] Bayman P. Mosquera-Espinosa A. T. Saladini-Aponte C. M. Hurtado-Guevara N. C. Viera-Ruiz N. L. (2016). Age-dependent mycorrhizal specificity in an invasive orchid, *Oeceoclades maculata*. Am. J. Bot. 103, 1880–1889. 10.3732/ajb.1600127, PMID: 27797713

[ref14] Bayman P. Otero J. T. (2006). “Microbial endophytes of orchid roots,” in Microbial Root Endophytes. eds. Schulz B. J. E. Boyle C. J. C. Sieber T. N. (Berlin, Heidelberg: Springer), 153–177.

[ref15] Bell J. Yokoya K. Kendon J. P. Sarasan V. (2020). Diversity of root-associated culturable fungi of *Cephalanthera rubra* (Orchidaceae) in relation to soil characteristics. PeerJ 8:e8695. 10.7717/peerj.8695, PMID: 32175192 PMC7058101

[ref16] Bellino A. Alfani A. Selosse M.-A. Guerrieri R. Borghetti M. Baldantoni D. (2014). Nutritional regulation in mixotrophic plants: new insights from *Limodorum abortivum*. Oecologia 175, 875–885. 10.1007/s00442-014-2940-8, PMID: 24817196

[ref17] Beltrán-Nambo M. Martínez-Trujillo M. Montero-Castro J. C. Salgado-Garciglia R. Otero-Ospina J. T. Carreón-Abud Y. (2018). Fungal diversity in the roots of four epiphytic orchids endemic to Southwest Mexico is related to the breadth of plant distribution. Rhizosphere 7, 49–56. 10.1016/j.rhisph.2018.07.001

[ref18] Bidartondo M. I. Bruns T. D. Weiß M. Sérgio C. Read D. J. (2003). Specialized cheating of the ectomycorrhizal symbiosis by an epiparasitic liverwort. Proc. R. Soc. B 270, 835–842. 10.1098/rspb.2002.2299, PMID: 12737662 PMC1691308

[ref19] Bidartondo M. I. Read D. J. (2008). Fungal specificity bottlenecks during orchid germination and development. Mol. Ecol. 17, 3707–3716. 10.1111/j.1365-294X.2008.03848.x, PMID: 18627452

[ref20] Binder M. Hibbett D. S. Larsson K. H. Larsson E. Langer E. Langer G. (2005). The phylogenetic distribution of resupinate forms across the major clades of mushroom-forming fungi (Homobasidiomycetes). Syst. Biodivers. 3, 113–157. 10.1017/S1477200005001623

[ref21] Bonfante P. Anca I. A. (2009). Plants, mycorrhizal fungi, and bacteria: a network of interactions. Annu. Rev. Microbiol. 63, 363–383. 10.1146/annurev.micro.091208.073504, PMID: 19514845

[ref22] Bonnardeaux Y. Brundrett M. Batty A. Dixon K. Koch J. Sivasithamparam K. (2007). Diversity of mycorrhizal fungi of terrestrial orchids: compatibility webs, brief encounters, lasting relationships and alien invasions. Mycol. Res. 111, 51–61. 10.1016/j.mycres.2006.11.006, PMID: 17289365

[ref23] Bougoure J. Ludwig M. Brundrett M. Grierson P. (2009). Identity and specificity of the fungi forming mycorrhizas with the rare mycoheterotrophic orchid *Rhizanthella gardneri*. Mycol. Res. 113, 1097–1106. 10.1016/j.mycres.2009.07.007, PMID: 19619652

[ref24] Brown S. P. Veach A. M. Rigdon-Huss A. R. Grond K. Lickteig S. K. Lothamer K.. (2015). Scraping the bottom of the barrel: are rare high throughput sequences artifacts? Fungal Ecol. 13, 221–225. 10.1016/j.funeco.2014.08.006

[ref25] Brundrett M. C. Tedersoo L. (2018). Evolutionary history of mycorrhizal symbioses and global host plant diversity. New Phytol. 220, 1108–1115. 10.1111/nph.14976, PMID: 29355963

[ref26] Calvert J. (2017). Mycorrhizal associations and phylogenetic relationships of South-east Queensland Bulbophyllum orchids. dissertation. Queensland: University of Southern Queensland.

[ref27] Cameron D. D. Johnson I. Read D. J. Leake J. R. (2008). Giving and receiving: measuring the carbon cost of mycorrhizas in the green orchid, *Goodyera repens*. New Phytol. 180, 176–184. 10.1111/j.1469-8137.2008.02533.x, PMID: 18627489

[ref28] Cardelús C. L. Colwell R. K. Watkins J. E. (2006). Vascular epiphyte distribution patterns: explaining the mid-elevation richness peak. J. Ecol. 94, 144–156. 10.1111/j.1365-2745.2005.01052.x

[ref29] Cevallos S. Declerck S. Suárez J. P. (2018b). *In situ* orchid seedling-trap experiment shows few keystone and many randomly-associated mycorrhizal fungal species during early plant colonization. Front. Plant Sci. 9:1664. 10.3389/fpls.2018.01664, PMID: 30505314 PMC6250785

[ref30] Cevallos S. Herrera P. Sánchez-Rodríguez A. Declerck S. Suárez J. P. (2018a). Untangling factors that drive community composition of root associated fungal endophytes of Neotropical epiphytic orchids. Fungal Ecol. 34, 67–75. 10.1016/j.funeco.2018.05.002

[ref31] Cevallos S. Sánchez-Rodríguez A. Decock C. Declerck S. Suárez J. P. (2017). Are there keystone mycorrhizal fungi associated to tropical epiphytic orchids? Mycorrhiza 27, 225–232. 10.1007/s00572-016-0746-8, PMID: 27882467

[ref32] Chase M. W. Cameron K. M. Freudenstein J. V. Pridgeon A. M. Salazar G. van den Berg C. (2015). An updated classification of Orchidaceae. Bot. J. Linn. Soc. 177, 151–174. 10.1111/boj.12234

[ref33] Chen Q. L. Ding J. Zhu D. Hu H. W. Delgado-Baquerizo M. Ma Y. B.. (2019). Rare microbial taxa as the major drivers of ecosystem multifunctionality in long-term fertilized soils. Soil Biol. Biochem. 141:107686. 10.1016/j.soilbio.2019.107686

[ref34] Claro A. Mujica M. I. Cisternas M. Armesto J. J. Pérez F. (2020). Low mycorrhizal diversity in the endangered and rare orchids *Bipinnula volckmannii* and *B. apinnula* of Central Chile. Symbiosis 80, 145–154. 10.1007/s13199-019-00648-w

[ref35] Clements M. A. (1988). Orchid mycorrhizal associations. Lindleyana 3, 73–86.

[ref36] Cordovez V. Dini-Andreote F. Carrión V. J. Raaijmakers J. M. (2019). Ecology and evolution of plant microbiomes. Annu. Rev. Microbiol. 73, 69–88. 10.1146/annurev-micro-090817-062524, PMID: 31091418

[ref37] Cruz D. Suárez J. P. Kottke I. Piepenbring M. (2014). Cryptic species revealed by molecular phylogenetic analysis of sequences obtained from basidiomata of *Tulasnella*. Mycologia 106, 708–722. 10.3852/12-386, PMID: 24874921

[ref38] Cruz D. Suárez J. P. Kottke I. Piepenbring M. Oberwinkler F. (2011). Defining species in *Tulasnella* by correlating morphology and nrDNA ITS-5.8S sequence data of basidiomata from a tropical Andean forest. Mycol. Prog. 10, 229–238. 10.1007/s11557-010-0692-3

[ref39] Davis B. J. Phillips R. D. Wright M. Linde C. C. Dixon K. W. (2015). Continent-wide distribution in mycorrhizal fungi: implications for the biogeography of specialized orchids. Ann. Bot. 116, 413–421. 10.1093/aob/mcv084, PMID: 26105186 PMC4549956

[ref40] Dearnaley J. D. W. (2007). Further advances in orchid mycorrhizal research. Mycorrhiza 17, 475–486. 10.1007/s00572-007-0138-1, PMID: 17582535

[ref41] Dearnaley J. D. W. Martos F. Selosse M.-A. (2012). “Orchid mycorrhizas: molecular ecology, physiology, evolution and conservation aspects,” in Fungal Associations. 2nd Edn. ed. Hock B. (Berlin, Heidelberg: Springer), 207–230.

[ref42] Dearnaley J. Perotto S. Selosse M.-A. (2016). “Structure and development of orchid mycorrhizas,” in Molecular Mycorrhizal Symbiosis. ed. Martin F. (Hoboken, NJ: John Wiley and Sons), 63–86.

[ref43] Diez J. M. (2007). Hierarchical patterns of symbiotic orchid germination linked to adult proximity and environmental gradients. J. Ecol. 95, 159–170. 10.1111/j.1365-2745.2006.01194.x

[ref44] Ding R. Chen X. H. Zhang L. J. Yu X. D. Qu B. Duan R.. (2014). Identity and specificity of *Rhizoctonia*-like fungi from different populations of *Liparis japonica* (Orchidaceae) in Northeast China. PLoS One 9:e105573. 10.1371/journal.pone.0105573, PMID: 25140872 PMC4139347

[ref45] Dini-Andreote F. Stegen J. C. van Elsas J. D. Salles J. F. (2015). Disentangling mechanisms that mediate the balance between stochastic and deterministic processes in microbial succession. Proc. Natl. Acad. Sci. U. S. A. 112, E1326–E1332. 10.1073/pnas.1414261112, PMID: 25733885 PMC4371938

[ref46] Downing J. L. Liu H. Shao S. C. Wang X. L. McCormick M. Deng R. Y.. (2017). Contrasting changes in biotic interactions of orchid populations subject to conservation introduction vs. conventional translocation in tropical China. Biol. Conserv. 212, 29–38. 10.1016/j.biocon.2017.05.021

[ref47] Duffy K. J. Waud M. Schatz B. Petanidou T. Jacquemyn H. (2019). Latitudinal variation in mycorrhizal diversity associated with a European orchid. J. Biogeogr. 46, 968–980. 10.1111/jbi.13548

[ref48] Egidi E. May T. W. Franks A. E. (2018). Seeking the needle in the haystack: undetectability of mycorrhizal fungi outside of the plant rhizosphere associated with an endangered Australian orchid. Fungal Ecol. 33, 13–23. 10.1016/j.funeco.2018.01.002

[ref49] Ercole E. Adamo M. Rodda M. Gebauer G. Girlanda M. Perotto S. (2015). Temporal variation in mycorrhizal diversity and carbon and nitrogen stable isotope abundance in the wintergreen meadow orchid *Anacamptis morio*. New Phytol. 205, 1308–1319. 10.1111/nph.13109, PMID: 25382295

[ref50] Esposito F. Jacquemyn H. Waud M. Tyteca D. (2016). Mycorrhizal fungal diversity and community composition in two closely related *Platanthera* (Orchidaceae) species. PLoS One 11:e0164108. 10.1371/journal.pone.0164108, PMID: 27695108 PMC5047478

[ref51] Fadiji A. E. Babalola O. O. (2020). Metagenomics methods for the study of plant-associated microbial communities: a review. J. Microbiol. Methods 170:105860. 10.1016/j.mimet.2020.105860, PMID: 32027927

[ref52] Favre-Godal Q. Gourguillon L. Lordel-Madeleine S. Gindro K. Choisy P. (2020). Orchids and their mycorrhizal fungi: an insufficiently explored relationship. Mycorrhiza 30, 5–22. 10.1007/s00572-020-00934-2, PMID: 31982950

[ref53] Fochi V. Chitarra W. Kohler A. Voyron S. Singan V. R. Lindquist E. A.. (2017). Fungal and plant gene expression in the *Tulasnella calospora*-*Serapias vomeracea* symbiosis provides clues about nitrogen pathways in orchid mycorrhizas. New Phytol. 213, 365–379. 10.1111/nph.14279, PMID: 27859287

[ref54] Gao C. Montoya L. Xu L. Madera M. Hollingsworth J. Purdom E.. (2020a). Fungal community assembly in drought-stressed sorghum shows stochasticity, selection, and universal ecological dynamics. Nat. Commun. 11:34. 10.1038/s41467-019-13913-9, PMID: 31911594 PMC6946711

[ref55] Gao Y. Zhao Z. Y. Li J. Y. Liu N. Jacquemyn H. Guo S. X.. (2020b). Do fungal associates of co-occurring orchids promote seed germination of the widespread orchid species *Gymnadenia conopsea*? Mycorrhiza 30, 221–228. 10.1007/s00572-020-00943-1, PMID: 32146514

[ref56] Gardes M. Bruns T. D. (1993). ITS primers with enhanced specificity for basidiomycetes-application to the identification of mycorrhizae and rusts. Mol. Ecol. 2, 113–118. 10.1111/j.1365-294X.1993.tb00005.x, PMID: 8180733

[ref57] Gebauer G. Preiss K. Gebauer A. C. (2016). Partial mycoheterotrophy is more widespread among orchids than previously assumed. New Phytol. 211, 11–15. 10.1111/nph.13865, PMID: 26832994

[ref58] Geml J. (2017). “Altitudinal gradients in mycorrhizal symbioses: The current state of knowledge on how richness and community structure change with elevation,” in Biogeography of Mycorrhizal Symbiosis: Ecological Studies. ed. Tedersoo L. (Switzerland, Cham: Springer), 107–123.

[ref59] Geml J. Pastor N. Fernandez L. Pacheco S. Semenova T. A. Becerra A. G.. (2014). Large-scale fungal diversity assessment in the Andean Yungas forests reveals strong community turnover among forest types along an altitudinal gradient. Mol. Ecol. 23, 2452–2472. 10.1111/mec.12765, PMID: 24762095

[ref60] Gerz M. Guillermo Bueno C. Ozinga W. A. Zobel M. Moora M. (2018). Niche differentiation and expansion of plant species are associated with mycorrhizal symbiosis. J. Ecol. 106, 254–264. 10.1111/1365-2745.12873

[ref61] Girlanda M. Segreto R. Cafasso D. Liebel H. T. Rodda M. Ercole E.. (2011). Photosynthetic Mediterranean meadow orchids feature partial mycoheterotrophy and specific mycorrhizal associations. Am. J. Bot. 98, 1148–1163. 10.3732/ajb.1000486, PMID: 21712419

[ref62] Girlanda M. Selosse M.-A. Cafasso D. Brilli F. Delfine S. Fabbian R.. (2006). Inefficient photosynthesis in the Mediterranean orchid *Limodorum abortivum* is mirrored by specific association to ectomycorrhizal Russulaceae. Mol. Ecol. 15, 491–504. 10.1111/j.1365-294X.2005.02770.x, PMID: 16448415

[ref63] Givnish T. J. Spalink D. Ames M. Lyon S. P. Hunter S. J. Zuluaga A.. (2015). Orchid phylogenomics and multiple drivers of their extraordinary diversification. Proc. R. Soc. B 282:20151553. 10.1098/rspb.2015.1553, PMID: 26311671 PMC4571710

[ref64] Givnish T. J. Spalink D. Ames M. Lyon S. P. Hunter S. J. Zuluaga A.. (2016). Orchid historical biogeography, diversification, Antarctica and the paradox of orchid dispersal. J. Biogeogr. 43, 1905–1916. 10.1111/jbi.12854

[ref65] González-Chávez M. C. A. Torres-Cruz T. J. Sánchez S. A. Carrillo-González R. Carrillo-López L. M. Porras-Alfaro A. (2018). Microscopic characterization of orchid mycorrhizal fungi: *Scleroderma* as a putative novel orchid mycorrhizal fungus of *Vanilla* in different crop systems. Mycorrhiza 28, 147–157. 10.1007/s00572-017-0808-6, PMID: 29177968

[ref66] Govinda Rajulu M. B. Suryanarayanan T. S. Tangjang S. (2016). Endophytic fungi of orchids of Arunachal Pradesh, north eastern India. Curr. Res. Environ. Appl. Mycol. 6, 293–299. 10.5943/cream/6/4/7

[ref67] Graham R. R. Dearnaley J. D. W. (2012). The rare Australian epiphytic orchid *Sarcochilus weinthalii* associates with a single species of *Ceratobasidium*[J]. Fungal Divers. 54, 31–37. 10.1007/s13225-011-0106-0

[ref68] Guo X. X. Zhang X. N. Qin Y. Liu Y. X. Zhang J. Y. Zhang N.. (2020). Host-associated quantitative abundance profiling reveals the microbial load variation of root microbiome. Plant Commun. 1:100003. 10.1016/j.xplc.2019.100003, PMID: 33404537 PMC7747982

[ref69] Han J. Y. Xiao H. F. Gao J. Y. (2016). Seasonal dynamics of mycorrhizal fungi in *Paphiopedilum spicerianum* (Rchb f) Pfitzer—a critically endangered orchid from China. Glob. Ecol. Conserv. 6, 327–338. 10.1016/j.gecco.2016.03.011

[ref71] Herrera H. García-Romera I. Meneses C. Pereira G. Arriagada C. (2019a). Orchid mycorrhizal interactions on the Pacific side of the Andes from Chile: a review. J. Soil Sci. Plant Nutr. 19, 187–202. 10.1007/s42729-019-00026-x

[ref72] Herrera P. Kottke I. Molina M. C. Méndez M. Suárez J. P. (2018). Generalism in the interaction of Tulasnellaceae mycobionts with orchids characterizes a biodiversity hotspot in the tropical Andes of southern Ecuador. Mycoscience 59, 38–48. 10.1016/j.myc.2017.08.003

[ref73] Herrera P. Suárez J. P. Sánchez-Rodríguez A. Molina M. C. Prieto M. Méndez M. (2019b). Many broadly-shared mycobionts characterize mycorrhizal interactions of two coexisting epiphytic orchids in a high elevation tropical forest. Fungal Ecol. 39, 26–36. 10.1016/j.funeco.2018.11.003

[ref74] Herrera-Rus I. Pastor J. E. Juan R. (2020). Fungal colonization associated with phenological stages of a photosynthetic terrestrial temperate orchid from the southern Iberian Peninsula. J. Plant Res. 133, 807–825. 10.1007/s10265-020-01225-9, PMID: 32968931

[ref75] Hoeksema J. D. Bever J. D. Chakraborty S. Bala Chaudhary V. Gardes M. Gehring C. A.. (2018). Evolutionary history of plant hosts and fungal symbionts predicts the strength of mycorrhizal mutualism. Commun. Biol. 1:116. 10.1038/s42003-018-0120-9, PMID: 30271996 PMC6123707

[ref76] Hu Y. J. Veresoglou S. D. Tedersoo L. Xu T. L. Ge T. D. Liu L.. (2019). Contrasting latitudinal diversity and co-occurrence patterns of soil fungi and plants in forest ecosystems. Soil Biol. Biochem. 131, 100–110. 10.1016/j.soilbio.2019.01.001

[ref77] Huynh T. T. Thomson R. Mclean C. B. Lawrie A. C. (2009). Functional and genetic diversity of mycorrhizal fungi from single plants of *Caladenia formosa* (Orchidaceae). Ann. Bot. 104, 757–765. 10.1093/aob/mcp153, PMID: 19561011 PMC2729648

[ref78] Hyde K. D. Soytong K. (2008). The fungal endophyte dilemma. Fungal Divers. 33, 163–173.

[ref79] Hyde K. D. Xu J. C. Rapior S. Jeewon R. Lumyong S. Niego A. G. T.. (2019). The amazing potential of fungi: 50 ways we can exploit fungi industrially. Fungal Divers. 97, 1–136. 10.1007/s13225-019-00430-9

[ref80] Hynson N. A. Madsen T. P. Selosse M.-A. Adam I. K. U. Ogura-Tsujita Y. Roy M.. (2013). “The physiological ecology of mycoheterotrophy,” in Mycoheterotrophy: The Biology of Plants Living on Fungi. ed. Merckx V. S. F. T. (New York, NY: Springer), 297–342.

[ref81] Illyés Z. Halász K. Rudnóy S. Ouanphanivanh N. Garay T. Bratek Z. (2009). Changes in the diversity of the mycorrhizal fungi of orchids as a function of the water supply of the habitat. J. Appl. Bot. Food Qual. 83, 28–36.

[ref82] Irwin M. J. Bougoure J. J. Dearnaley J. D. W. (2007). *Pterostylis nutans* (Orchidaceae) has a specific association with two *Ceratobasidium* root-associated fungi across its range in eastern Australia. Mycoscience 48, 231–239. 10.1007/s10267-007-0360-x

[ref83] Izuddin M. Srivathsan A. Lee A. L. Yam T. W. Webb E. L. (2019). Availability of orchid mycorrhizal fungi on roadside trees in a tropical urban landscape. Sci. Rep. 9:19528. 10.1038/s41598-019-56049-y, PMID: 31863015 PMC6925147

[ref84] Jacquemyn H. Brys R. Cammue B. P. A. Honnay O. Lievens B. (2011a). Mycorrhizal associations and reproductive isolation in three closely related *Orchis* species. Ann. Bot. 107, 347–356. 10.1093/aob/mcq248, PMID: 21186239 PMC3043927

[ref85] Jacquemyn H. Brys R. Lievens B. Wiegand T. (2012). Spatial variation in below-ground seed germination and divergent mycorrhizal associations correlate with spatial segregation of three co-occurring orchid species. J. Ecol. 100, 1328–1337. 10.1111/j.1365-2745.2012.01998.x

[ref86] Jacquemyn H. Brys R. Merckx V. S. F. T. Waud M. Lievens B. Wiegand T. (2014). Coexisting orchid species have distinct mycorrhizal communities and display strong spatial segregation. New Phytol. 202, 616–627. 10.1111/nph.12640, PMID: 24325257

[ref87] Jacquemyn H. Brys R. Waud M. Busschaert P. Lievens B. (2015a). Mycorrhizal networks and coexistence in species-rich orchid communities. New Phytol. 206, 1127–1134. 10.1111/nph.13281, PMID: 25614926

[ref88] Jacquemyn H. Brys R. Waud M. Evans A. Figura T. Selosse M.-A. (2021). Mycorrhizal communities and isotope signatures in two partially mycoheterotrophic orchids. Front. Plant Sci. 12:618140. 10.3389/fpls.2021.618140, PMID: 33633765 PMC7901878

[ref89] Jacquemyn H. Duffy K. J. Selosse M.-A. (2017a). “Biogeography of orchid mycorrhizas,” in Biogeography of Mycorrhizal Symbiosis: Ecological Studies. ed. Tedersoo L. (Switzerland, Cham: Springer), 159–177.

[ref90] Jacquemyn H. Honnay O. Cammue B. P. A. Brys R. Lievens B. (2010). Low specificity and nested subset structure characterize mycorrhizal associations in five closely related species of the genus *Orchis*. Mol. Ecol. 19, 4086–4095. 10.1111/j.1365-294X.2010.04785.x, PMID: 20735736

[ref91] Jacquemyn H. Merckx V. S. F. T. (2019). Mycorrhizal symbioses and the evolution of trophic modes in plants. J. Ecol. 107, 1567–1581. 10.1111/1365-2745.13165

[ref92] Jacquemyn H. Merckx V. Brys R. Tyteca D. Cammue B. P. A. Honnay O.. (2011b). Analysis of network architecture reveals phylogenetic constraints on mycorrhizal specificity in the genus *Orchis* (Orchidaceae). New Phytol. 192, 518–528. 10.1111/j.1469-8137.2011.03796.x, PMID: 21668874

[ref93] Jacquemyn H. Waud M. Brys R. Lallemand F. Courty P. E. Robionek A.. (2017b). Mycorrhizal associations and trophic modes in coexisting orchids: an ecological continuum between auto- and mixotrophy. Front. Plant Sci. 8:1497. 10.3389/fpls.2017.01497, PMID: 28912791 PMC5583604

[ref94] Jacquemyn H. Waud M. Lievens B. Brys R. (2016a). Differences in mycorrhizal communities between *Epipactis palustris*, *E. helleborine* and its presumed sister species *E. neerlandica*. Ann. Bot. 118, 105–114. 10.1093/aob/mcw015, PMID: 26946528 PMC4934391

[ref95] Jacquemyn H. Waud M. Merckx V. S. F. T. Brys R. Tyteca D. Hedrén M.. (2016b). Habitat-driven variation in mycorrhizal communities in the terrestrial orchid genus *Dactylorhiza*. Sci. Rep. 6:37182. 10.1038/srep37182, PMID: 27883008 PMC5121631

[ref96] Jacquemyn H. Waud M. Merckx V. S. F. T. Lievens B. Brys R. (2015b). Mycorrhizal diversity, seed germination and long-term changes in population size across nine populations of the terrestrial orchid *Neottia ovata*. Mol. Ecol. 24, 3269–3280. 10.1111/mec.13236, PMID: 25963669

[ref97] Jasinge N. U. Huynh T. Lawrie A. C. (2018). Changes in orchid populations and endophytic fungi with rainfall and prescribed burning in *Pterostylis revoluta* in Victoria, Australia. Ann. Bot. 121, 321–334. 10.1093/aob/mcx164, PMID: 29300863 PMC5808809

[ref98] Ji B. W. Sheth R. U. Dixit P. D. Huang Y. M. Kaufman A. Wang H. H.. (2019). Quantifying spatiotemporal variability and noise in absolute microbiota abundances using replicate sampling. Nat. Methods 16, 731–736. 10.1038/s41592-019-0467-y, PMID: 31308552 PMC7219825

[ref99] Jiang J. W. Zhang K. Cheng S. Nie Q. W. Zhou S. X. Chen Q. Q.. (2019). *Fusarium oxysporum* KB-3 from *Bletilla striata*: an orchid mycorrhizal fungus. Mycorrhiza 29, 531–540. 10.1007/s00572-019-00904-3, PMID: 31270609

[ref100] Jousset A. Bienhold C. Chatzinotas A. Gallien L. Gobet A. Kurm V.. (2017). Where less may be more: how the rare biosphere pulls ecosystems strings. ISME J. 11, 853–862. 10.1038/ismej.2016.174, PMID: 28072420 PMC5364357

[ref101] Julou T. Burghardt B. Gebauer G. Berveiller D. Damesin C. Selosse M.-A. (2005). Mixotrophy in orchids: insights from a comparative study of green individuals and nonphotosynthetic individuals of *Cephalanthera damasonium*. New Phytol. 166, 639–653. 10.1111/j.1469-8137.2005.01364.x, PMID: 15819926

[ref102] Kartzinel T. R. Trapnell D. W. Shefferson R. P. (2013). Highly diverse and spatially heterogeneous mycorrhizal symbiosis in a rare epiphyte is unrelated to broad biogeographic or environmental features. Mol. Ecol. 22, 5949–5961. 10.1111/mec.12536, PMID: 24112555

[ref103] Kaur J. Andrews L. Sharma J. (2019). High specificity of a rare terrestrial orchid toward a rare fungus within the north American tallgrass prairie. Fungal Biol. 123, 895–904. 10.1016/j.funbio.2019.09.010, PMID: 31733732

[ref104] Kinoshita A. Ogura-Tsujita Y. Umata H. Sato H. Hashimoto T. Yukawa T. (2016). How do fungal partners affect the evolution and habitat preferences of mycoheterotrophic plants? A case study in *Gastrodia*. Am. J. Bot. 103, 207–220. 10.3732/ajb.1500082, PMID: 26838365

[ref105] Kohler A. Kuo A. Nagy L. G. Morin E. Barry K. W. Buscot F.. (2015). Convergent losses of decay mechanisms and rapid turnover of symbiosis genes in mycorrhizal mutualists. Nat. Genet. 47, 410–415. 10.1038/ng.3223, PMID: 25706625

[ref106] Köhler J. Yang N. Pena R. Raghavan V. Polle A. Meier I. C. (2018). Ectomycorrhizal fungal diversity increases phosphorus uptake efficiency of European beech. New Phytol. 220, 1200–1210. 10.1111/nph.15208, PMID: 29770963

[ref107] Kohout P. Těšitelová T. Roy M. Vohník M. Jersáková J. (2013). A diverse fungal community associated with *Pseudorchis albida* (Orchidaceae) roots. Fungal Ecol. 6, 50–64. 10.1016/j.funeco.2012.08.005

[ref108] Kottke I. Setaro S. Haug I. Herrera P. Cruz D. Fries A.. (2013). “Mycorrhiza networks promote biodiversity and stabilize the tropical mountain rain forest ecosystem: perspectives for understanding complex communities,” in Ecosystem Services, Biodiversity and Environmental Change in a Tropical Mountain Ecosystem of South Ecuador: Ecological Studies. eds. Bendix J. Beck E. Bräuning A. Makeschin F. Mosandl R. Scheu S.. (Berlin, Heidelberg: Springer), 187–203.

[ref109] Kottke I. Suárez J. P. Herrera P. Cruz D. Bauer R. Haug I.. (2010). Atractiellomycetes belonging to the ‘rust’ lineage (Pucciniomycotina) form mycorrhizae with terrestrial and epiphytic neotropical orchids. Proc. R. Soc. B 277, 1289–1298. 10.1098/rspb.2009.1884, PMID: 20007181 PMC2842812

[ref110] Küper W. Kreft H. Nieder J. Köster N. Barthlott W. (2004). Large-scale diversity patterns of vascular epiphytes in Neotropical montane rain forests. J. Biogeogr. 31, 1477–1487. 10.1111/j.1365-2699.2004.01093.x

[ref111] Lallemand F. Logacheva M. Clainche I. L. Bérard A. Zheleznaia E. May M.. (2019). Thirteen new plastid genomes from mixotrophic and autotrophic species provide insights into heterotrophy evolution in Neottieae orchids. Genome Biol. Evol. 11, 2457–2467. 10.1093/gbe/evz170, PMID: 31396616 PMC6733356

[ref112] Lee Y. I. Yang C. K. Gebauer G. (2015). The importance of associations with saprotrophic non-*Rhizoctonia* fungi among fully mycoheterotrophic orchids is currently under-estimated: novel evidence from sub-tropical Asia. Ann. Bot. 116, 423–435. 10.1093/aob/mcv085, PMID: 26113634 PMC4549957

[ref113] Liang Y. T. Xiao X. Nuccio E. E. Yuan M. T. Zhang N. Xue K.. (2020). Differentiation strategies of soil rare and abundant microbial taxa in response to changing climatic regimes. Environ. Microbiol. 22, 1327–1340. 10.1111/1462-2920.14945, PMID: 32067386

[ref114] Lindahl B. D. Nilsson R. H. Tedersoo L. Abarenkov K. Carlsen T. Kjøller R.. (2013). Fungal community analysis by high-throughput sequencing of amplified markers—a user’s guide. New Phytol. 199, 288–299. 10.1111/nph.12243, PMID: 23534863 PMC3712477

[ref115] Liu Q. Chen J. Corlett R. T. Fan X. L. Yu D. L. Yang H. P.. (2015a). Orchid conservation in the biodiversity hotspot of southwestern China. Conserv. Biol. 29, 1563–1572. 10.1111/cobi.12584, PMID: 26372504

[ref116] Liu T. L. Li C. M. Han Y. L. Chiang T. Y. Chiang Y. C. Sung H. M. (2015b). Highly diversified fungi are associated with the achlorophyllous orchid *Gastrodia flavilabella*. BMC Genomics 16:185. 10.1186/s12864-015-1422-7, PMID: 25886817 PMC4371811

[ref117] Liu H. Liu Z. J. Jin X. H. Gao J. Y. Chen Y. Liu Q.. (2020). Assessing conservation efforts against threats to wild orchids in China. Biol. Conserv. 243:108484. 10.1016/j.biocon.2020.108484

[ref118] Liu Y. X. Qin Y. Bai Y. (2019). Reductionist synthetic community approaches in root microbiome research. Curr. Opin. Microbiol. 49, 97–102. 10.1016/j.mib.2019.10.010, PMID: 31734603

[ref119] Ma X. Y. Kang J. C. Nontachaiyapoom S. Wen T. C. Hyde K. D. (2015). Non-mycorrhizal endophytic fungi from orchids. Curr. Sci. 109, 72–87.

[ref120] Martos F. Dulormne M. Pailler T. Bonfante P. Faccio A. Fournel J.. (2009). Independent recruitment of saprotrophic fungi as mycorrhizal partners by tropical achlorophyllous orchids. New Phytol. 184, 668–681. 10.1111/j.1469-8137.2009.02987.x, PMID: 19694964

[ref121] Martos F. Munoz F. Pailler T. Kottke I. Gonneau C. Selosse M.-A. (2012). The role of epiphytism in architecture and evolutionary constraint within mycorrhizal networks of tropical orchids. Mol. Ecol. 21, 5098–5109. 10.1111/j.1365-294X.2012.05692.x, PMID: 22765763

[ref122] May M. Jąkalski M. Novotná A. Dietel J. Ayasse M. Lallemand F.. (2020). Three-year pot culture of *Epipactis helleborine* reveals autotrophic survival, without mycorrhizal networks, in a mixotrophic species. Mycorrhiza 30, 51–61. 10.1007/s00572-020-00932-4, PMID: 31965295

[ref123] McCormick M. K. Jacquemyn H. (2014). What constrains the distribution of orchid populations? New Phytol. 202, 392–400. 10.1111/nph.12639

[ref124] McCormick M. K. Taylor D. L. Juhaszova K. Burnett R. K. Whigham D. F. O’Neill J. P. (2012). Limitations on orchid recruitment: not a simple picture. Mol. Ecol. 21, 1511–1523. 10.1111/j.1365-294X.2012.05468.x, PMID: 22272942

[ref125] McCormick M. K. Taylor D. L. Whigham D. F. Burnett R. K. (2016). Germination patterns in three terrestrial orchids relate to abundance of mycorrhizal fungi. J. Ecol. 104, 744–754. 10.1111/1365-2745.12556

[ref126] McCormick M. K. Whigham D. F. Canchani-Viruet A. (2018). Mycorrhizal fungi affect orchid distribution and population dynamics. New Phytol. 219, 1207–1215. 10.1111/nph.15223, PMID: 29790578

[ref127] McCormick M. K. Whigham D. F. O’Neill J. P. Becker J. J. Werner S. Rasmussen H. N.. (2009). Abundance and distribution of *Corallorhiza odontorhiza* reflect variations in climate and ectomycorrhizae. Ecol. Monogr. 79, 619–635. 10.1890/08-0729.1

[ref128] McCormick M. K. Whigham D. F. Sloan D. O’Malley K. Hodkinson B. (2006). Orchid-fungus fidelity: a marriage meant to last? Ecology 87, 903–911. 10.1890/0012-9658(2006)87[903,OFAMMT]2.0.CO;2, PMID: 16676534

[ref129] McKendrick S. L. Leake J. R. Read D. J. (2000). Symbiotic germination and development of myco-heterotrophic plants in nature: transfer of carbon from ectomycorrhizal *Salix repens* and *Betula pendula* to the orchid *Corallorhiza trifida* through shared hyphal connections. New Phytol. 145, 539–548. 10.1046/j.1469-8137.2000.00592.x, PMID: 33862911

[ref130] McKendrick S. L. Leake J. R. Taylor D. L. Read D. J. (2002). Symbiotic germination and development of the myco-heterotrophic orchid *Neottia nidus-avis* in nature and its requirement for locally distributed *Sebacina* spp. New Phytol. 154, 233–247. 10.1046/j.1469-8137.2002.00372.x

[ref131] Mello A. Napoli C. Murat C. Morin E. Marceddu G. Bonfante P. (2011). ITS-1 versus ITS-2 pyrosequencing: a comparison of fungal populations in truffle grounds. Mycologia 103, 1184–1193. 10.3852/11-027, PMID: 21700633

[ref132] Meng Y. Y. Fan X. L. Zhou L. R. Shao S. C. Liu Q. Selosse M.-A.. (2019a). Symbiotic fungi undergo a taxonomic and functional bottleneck during orchid seeds germination: a case study on *Dendrobium moniliforme*. Symbiosis 79, 205–212. 10.1007/s13199-019-00647-x

[ref133] Meng Y. Y. Shao S. C. Liu S. J. Gao J. Y. (2019b). Do the fungi associated with roots of adult plants support seed germination? A case study on *Dendrobium exile* (Orchidaceae). Glob. Ecol. Conserv. 17:e00582. 10.1016/j.gecco.2019.e00582

[ref134] Meng Y. Y. Zhang W. L. Selosse M.-A. Gao J. Y. (2019c). Are fungi from adult orchid roots the best symbionts at germination? A case study. Mycorrhiza 29, 541–547. 10.1007/s00572-019-00907-0, PMID: 31312918

[ref135] Merckx V. S. F. T. (2013). “Mycoheterotrophy: an introduction,” in Mycoheterotrophy: The Biology of Plants Living on Fungi. ed. Merckx V. S. F. T. (New York, NY: Springer), 1–17.

[ref136] Miura C. Yamaguchi K. Miyahara R. Yamamoto T. Fuji M. Yagame T.. (2018). The mycoheterotrophic symbiosis between orchids and mycorrhizal fungi possesses major components shared with mutualistic plant-mycorrhizal symbioses. Mol. Plant-Microbe Interact. 31, 1032–1047. 10.1094/MPMI-01-18-0029-R, PMID: 29649962

[ref137] Mujica M. I. Pérez M. F. Jakalski M. Martos F. Selosse M.-A. (2020). Soil P reduces mycorrhizal colonization while favors fungal pathogens: observational and experimental evidence in *Bipinnula* (Orchidaceae). FEMS Microbiol. Ecol. 96:fiaa178. 10.1093/femsec/fiaa178, PMID: 32845297

[ref138] Mujica M. I. Saez N. Cisternas M. Manzano M. Armesto J. J. Pérez F. (2016). Relationship between soil nutrients and mycorrhizal associations of two *Bipinnula* species (Orchidaceae) from Central Chile. Ann. Bot. 118, 149–158. 10.1093/aob/mcw082, PMID: 27311572 PMC4934401

[ref139] Müller D. B. Vogel C. Bai Y. Vorholt J. A. (2016). The plant microbiota: systems-level insights and perspectives. Annu. Rev. Genet. 50, 211–234. 10.1146/annurev-genet-120215-034952, PMID: 27648643

[ref140] Newsham K. K. (2011). A meta-analysis of plant responses to dark septate root endophytes. New Phytol. 190, 783–793. 10.1111/j.1469-8137.2010.03611.x, PMID: 21244432

[ref141] Nilsson R. H. Anslan S. Bahram M. Wurzbacher C. Baldrian P. Tedersoo L. (2019). Mycobiome diversity: high-throughput sequencing and identification of fungi. Nat. Rev. Microbiol. 17, 95–109. 10.1038/s41579-018-0116-y, PMID: 30442909

[ref142] Novotná A. Benítez Á. Herrera P. Cruz D. Filipczyková E. Suárez J. P. (2018). High diversity of root-associated fungi isolated from three epiphytic orchids in southern Ecuador. Mycoscience 59, 24–32. 10.1016/j.myc.2017.07.007

[ref143] Nurfadilah S. Swarts N. D. Dixon K. W. Lambers H. Merritt D. J. (2013). Variation in nutrient-acquisition patterns by mycorrhizal fungi of rare and common orchids explains diversification in a global biodiversity hotspot. Ann. Bot. 111, 1233–1241. 10.1093/aob/mct064, PMID: 23532043 PMC3662510

[ref144] Oberwinkler F. Cruz D. Suárez J. P. (2017). “Biogeography and ecology of Tulasnellaceae,” in Biogeography of Mycorrhizal Symbiosis: Ecological Studies. ed. Tedersoo L. (Switzerland, Cham: Springer), 237–271.

[ref145] Ogura-Tsujita Y. Gebauer G. Xu H. Fukasawa Y. Umata H. Tetsuka K.. (2018). The giant mycoheterotrophic orchid *Erythrorchis altissima* is associated mainly with a divergent set of wood-decaying fungi. Mol. Ecol. 27, 1324–1337. 10.1111/mec.14524, PMID: 29419910

[ref146] Oja J. Kohout P. Tedersoo L. Kull T. Kõljalg U. (2015). Temporal patterns of orchid mycorrhizal fungi in meadows and forests as revealed by 454 pyrosequencing. New Phytol. 205, 1608–1618. 10.1111/nph.13223, PMID: 25546739

[ref147] Oja J. Vahtra J. Bahram M. Kohout P. Kull T. Rannap R.. (2017). Local-scale spatial structure and community composition of orchid mycorrhizal fungi in semi-natural grasslands. Mycorrhiza 27, 355–367. 10.1007/s00572-016-0755-7, PMID: 28039600

[ref148] Oktalira F. T. Whitehead M. R. Linde C. C. (2019). Mycorrhizal specificity in widespread and narrow-range distributed *Caladenia* orchid species. Fungal Ecol. 42:100869. 10.1016/j.funeco.2019.100869

[ref149] Oliveira S. F. Bocayuva M. F. Veloso T. G. R. Bazzolli D. M. S. da Silva C. C. Pereira O. L.. (2014). Endophytic and mycorrhizal fungi associated with roots of endangered native orchids from the Atlantic Forest, Brazil. Mycorrhiza 24, 55–64. 10.1007/s00572-013-0512-0, PMID: 23812655

[ref150] Paduano C. Rodda M. Ercole E. Girlanda M. Perotto S. (2011). Pectin localization in the Mediterranean orchid *Limodorum abortivum* reveals modulation of the plant interface in response to different mycorrhizal fungi. Mycorrhiza 21, 97–104. 10.1007/s00572-010-0315-5, PMID: 20428900

[ref151] Pandey M. Sharma J. Taylor D. L. Yadon V. L. (2013). A narrowly endemic photosynthetic orchid is non-specific in its mycorrhizal associations. Mol. Ecol. 22, 2341–2354. 10.1111/mec.12249, PMID: 23432406

[ref152] Peay K. G. Kennedy P. G. Bruns T. D. (2011). Rethinking ectomycorrhizal succession: are root density and hyphal exploration types drivers of spatial and temporal zonation? Fungal Ecol. 4, 233–240. 10.1016/j.funeco.2010.09.010

[ref153] Pecoraro L. Caruso T. Cai L. Gupta V. K. Liu Z. J. (2018). Fungal networks and orchid distribution: new insights from above-and below-ground analyses of fungal communities. IMA Fungus 9, 1–11. 10.5598/imafungus.2018.09.01.01, PMID: 30018868 PMC6048571

[ref154] Pecoraro L. Huang L. Q. Caruso T. Perotto S. Girlanda M. Cai L.. (2017). Fungal diversity and specificity in *Cephalanthera damasonium* and *C. longifolia* (Orchidaceae) mycorrhizas. J. Syst. Evol. 55, 158–169. 10.1111/jse.12238

[ref155] Phillips R. D. Barrett M. D. Dalziell E. L. Dixon K. W. Swarts N. D. (2016). Geographical range and host breadth of *Sebacina* orchid mycorrhizal fungi associating with *Caladenia* in South-Western Australia. Bot. J. Linn. Soc. 182, 140–151. 10.1111/boj.12453

[ref156] Phillips R. D. Barrett M. D. Dixon K. W. Hopper S. D. (2011). Do mycorrhizal symbioses cause rarity in orchids? J. Ecol. 99, 858–869. 10.1111/j.1365-2745.2011.01797.x

[ref157] Pickles B. J. Genney D. R. Potts J. M. Lennon J. J. Anderson I. C. Alexander I. J. (2010). Spatial and temporal ecology of scots pine ectomycorrhizas. New Phytol. 186, 755–768. 10.1111/j.1469-8137.2010.03204.x, PMID: 20202132

[ref158] Pinto-Carbó M. Sieber S. Dessein S. Wicker T. Verstraete B. Gademann K.. (2016). Evidence of horizontal gene transfer between obligate leaf nodule symbionts. ISME J. 10, 2092–2105. 10.1038/ismej.2016.27, PMID: 26978165 PMC4989318

[ref159] Põlme S. Bahram M. Jacquemyn H. Kennedy P. Kohout P. Moora M.. (2018). Host preference and network properties in biotrophic plant-fungal associations. New Phytol. 217, 1230–1239. 10.1111/nph.14895, PMID: 29165806

[ref160] Props R. Kerckhof F. M. Rubbens P. Vrieze J. D. Sanabria E. H. Waegeman W.. (2017). Absolute quantification of microbial taxon abundances. ISME J. 11, 584–587. 10.1038/ismej.2016.117, PMID: 27612291 PMC5270559

[ref161] Qin J. Zhang W. Ge Z. W. Zhang S. B. (2019). Molecular identifications uncover diverse fungal symbionts of *Pleione* (Orchidaceae). Fungal Ecol. 37, 19–29. 10.1016/j.funeco.2018.10.003

[ref162] Rammitsu K. Yagame T. Yamashita Y. Yukawa T. Isshiki S. Ogura-Tsujita Y. (2019). A leafless epiphytic orchid, *Taeniophyllum glandulosum* Blume (Orchidaceae), is specifically associated with the Ceratobasidiaceae family of basidiomycetous fungi. Mycorrhiza 29, 159–166. 10.1007/s00572-019-00881-7, PMID: 30707331

[ref163] Ramsay R. R. Sivasithamparam K. Dixon K. W. (1987). Anastomosis groups among *Rhizoctonia*-like endophytic fungi in southwestern Australian *Pterostylis* species (Orchidaceae). Lindleyana 2, 161–166.

[ref164] Ran Y. Z. Xu J. T. (1988). Studies on the inhibition of seed germination of Gastrodia elata BI. By *Armillaria mellea* Qul. Zhong Yao Tong Bao 13, 15–17. 62. PMID: 3252984

[ref165] Rasmussen H. N. (1995). Terrestrial Orchids: From Seed to Mycotrophic Plant. New York, NY: Cambridge University Press.

[ref166] Rasmussen H. N. Rasmussen F. N. (2018). The epiphytic habitat on a living host: reflections on the orchid-tree relationship. Bot. J. Linn. Soc. 186, 456–472. 10.1093/botlinnean/box085

[ref167] Rasmussen H. N. Whigham D. F. (2002). Phenology of roots and mycorrhiza in orchid species differing in phototrophic strategy. New Phytol. 154, 797–807. 10.1046/j.1469-8137.2002.00422.x 33873451

[ref168] Raza M. Zhang Z. F. Hyde K. D. Diao Y. Z. Cai L. (2019). Culturable plant pathogenic fungi associated with sugarcane in southern China. Fungal Divers. 99, 1–104. 10.1007/s13225-019-00434-5

[ref169] Reiter N. Phillips R. D. Swarts N. D. Wright M. Holmes G. Sussmilch F. C.. (2020). Specific mycorrhizal associations involving the same fungal taxa in common and threatened *Caladenia* (Orchidaceae): implications for conservation. Ann. Bot. 126, 943–955. 10.1093/aob/mcaa116, PMID: 32574356 PMC7539350

[ref170] Richard F. Millot S. Gardes M. Selosse M.-A. (2005). Diversity and structuration by hosts of the below-ground mycorrhizal community in an old-growth Mediterranean forest dominated by *Quercus ilex* L. New Phytol. 166, 1011–1023. 10.1111/j.1469-8137.2005.01382.x, PMID: 15869659

[ref171] Roche S. A. Carter R. J. Peakall R. Smith L. M. Whitehead M. R. Linde C. C. (2010). A narrow group of monophyletic *Tulasnella* (Tulasnellaceae) symbiont lineages are associated with multiple species of *Chiloglottis* (Orchidaceae): implications for orchid diversity. Am. J. Bot. 97, 1313–1327. 10.3732/ajb.1000049, PMID: 21616884

[ref172] Rodriguez R. J. White J. F. Jr. Arnold A. E. Redman R. S. (2009). Fungal endophytes: diversity and functional roles. New Phytol. 182, 314–330. 10.1111/j.1469-8137.2009.02773.x, PMID: 19236579

[ref173] Roy M. Gonneau C. Rocheteau A. Berveiller D. Thomas J. C. Damesin C.. (2013). Why do mixotrophic plants stay green? A comparison between green and achlorophyllous orchid individuals *in situ*. Ecol. Monogr. 83, 95–117. 10.1890/11-2120.1

[ref174] Salazar J. M. Pomavilla M. Pollard A. T. Chica E. J. Peña D. F. (2020). Endophytic fungi associated with roots of epiphytic orchids in two Andean forests in southern Ecuador and their role in germination. Lankesteriana 20, 37–47. 10.15517/LANK.V20I1.41157

[ref175] Salazar-Cerezo S. Martinez-Montiel N. Cruz-Lopez M. C. Martinez-Contreras R. D. (2018). Fungal diversity and community composition of culturable fungi in *Stanhopea trigrina* cast gibberellin producers. Front. Microbiol. 9:612. 10.3389/fmicb.2018.00612, PMID: 29670591 PMC5893766

[ref176] Sarsaiya S. Jain A. Jia Q. Fan X. K. Shu F. X. Chen Z. W.. (2020). Molecular identification of endophytic fungi and their pathogenicity evaluation against *Dendrobium nobile* and *Dendrobium officinale*. Int. J. Mol. Sci. 21:316. 10.3390/ijms21010316, PMID: 31906579 PMC6982089

[ref177] Sarsaiya S. Shi J. S. Chen J. S. (2019). A comprehensive review on fungal endophytes and its dynamics on Orchidaceae plants: current research, challenges, and future possibilities. Bioengineered 10, 316–334. 10.1080/21655979.2019.1644854, PMID: 31347943 PMC6682353

[ref178] Sathiyadash K. Muthukumar T. Uma E. Pandey R. R. (2012). Mycorrhizal association and morphology in orchids. J. Plant Interact. 7, 238–247. 10.1080/17429145.2012.699105

[ref179] Schatz B. Geoffroy A. Dainat B. Bessière J. M. Buatois B. Hossaert-McKey M.. (2010). A case study of modified interactions with symbionts in a hybrid mediterranean orchid. Am. J. Bot. 97, 1278–1288. 10.3732/ajb.0900303, PMID: 21616880

[ref180] Schiebold J. M. I. Bidartondo M. I. Lenhard F. Makiola A. Gebauer G. (2018). Exploiting mycorrhizas in broad daylight: partial mycoheterotrophy is a common nutritional strategy in meadow orchids. J. Ecol. 106, 168–178. 10.1111/1365-2745.12831

[ref181] Schweiger J. M. I. Bidartondo M. I. Gebauer G. (2018). Stable isotope signatures of underground seedlings reveal the organic matter gained by adult orchids from mycorrhizal fungi. Funct. Ecol. 32, 870–881. 10.1111/1365-2435.13042

[ref182] Selosse M.-A. (2014). The latest news from biological interactions in orchids: in love, head to toe. New Phytol. 202, 337–340. 10.1111/nph.12769, PMID: 24645780

[ref183] Selosse M.-A. Boullard B. Richardson D. (2011). Noël Bernard (1874–1911): orchids to symbiosis in a dozen years, one century ago. Symbiosis 54, 61–68. 10.1007/s13199-011-0131-5

[ref184] Selosse M.-A. Faccio A. Scappaticci G. Bonfante P. (2004). Chlorophyllous and achlorophyllous specimens of *Epipactis microphylla* (Neottieae, Orchidaceae) are associated with ectomycorrhizal septomycetes, including truffles. Microb. Ecol. 47, 416–426. 10.1007/s00248-003-2034-3, PMID: 15107957

[ref185] Selosse M.-A. Martos F. (2014). Do chlorophyllous orchids heterotrophically use mycorrhizal fungal carbon? Trends Plant Sci. 19, 683–685. 10.1016/j.tplants.2014.09.005, PMID: 25278267

[ref186] Selosse M.-A. Martos F. Perry B. Maj P. Roy M. Pailler T. (2010). Saprotrophic fungal symbionts in tropical achlorophyllous orchids: finding treasures among the ‘molecular scraps’? Plant Signal. Behav. 5, 1–5. 10.4161/psb.5.4.10791, PMID: 20061806 PMC2958584

[ref187] Selosse M.-A. Schneider-Maunoury L. Martos F. (2018). Time to re-think fungal ecology? Fungal ecological niches are often prejudged. New Phytol. 217, 968–972. 10.1111/nph.14983, PMID: 29334598

[ref188] Selosse M.-A. Tacon F. L. (1998). The land flora: a phototroph-fungus partnership? Trends Ecol. Evol. 13, 15–20. 10.1016/S0169-5347(97)01230-5, PMID: 21238179

[ref189] Selosse M.-A. WEIss M. Jany J. L. Tillier A. (2002). Communities and populations of sebacinoid basidiomycetes associated with the achlorophyllous orchid *Neottia nidus-avis* (L.) L.C.M. Rich. and neighbouring tree ectomycorrhizae. Mol. Ecol. 11, 1831–1844. 10.1046/j.1365-294X.2002.01553.x, PMID: 12207732

[ref190] Shah S. Shrestha R. Maharjan S. Selosse M.-A. Pant B. (2019). Isolation and characterization of plant growth-promoting endophytic fungi from the roots of *Dendrobium moniliforme*. Plan. Theory 8:5. 10.3390/plants8010005, PMID: 30597827 PMC6359427

[ref191] Shefferson R. P. Bunch W. Cowden C. C. Lee Y. I. Kartzinel T. R. Yukawa T.. (2019). Does evolutionary history determine specificity in broad ecological interactions? J. Ecol. 107, 1582–1593. 10.1111/1365-2745.13170

[ref192] Shefferson R. P. Cowden C. C. McCormick M. K. Yukawa T. Ogura-Tsujita Y. Hashimoto T. (2010). Evolution of host breadth in broad interactions: mycorrhizal specificity in east Asian and north American rattlesnake plantains (*Goodyera* spp.) and their fungal hosts. Mol. Ecol. 19, 3008–3017. 10.1111/j.1365-294X.2010.04693.x, PMID: 20584135

[ref193] Shefferson R. P. Kull T. Hutchings M. J. Selosse M.-A. Jacquemyn H. Kellett K. M.. (2018). Drivers of vegetative dormancy across herbaceous perennial plant species. Ecol. Lett. 21, 724–733. 10.1111/ele.12940, PMID: 29575384

[ref194] Shefferson R. P. Taylor D. L. Weiß M. Garnica S. McCormick M. K. Adams S.. (2007). The evolutionary history of mycorrhizal specificity among lady’s slipper orchids. Evolution 61, 1380–1390. 10.1111/j.1558-5646.2007.00112.x, PMID: 17542847

[ref195] Shefferson R. P. Weiß M. Kull T. Taylor D. L. (2005). High specificity generally characterizes mycorrhizal association in rare lady’s slipper orchids, genus *Cypripedium*. Mol. Ecol. 14, 613–626. 10.1111/j.1365-294X.2005.02424.x, PMID: 15660950

[ref196] Sisti L. S. Flores-Borges D. N. A. Andrade S. A. L. Koehler S. Bonatelli M. L. Mayer J. L. S. (2019). The role of non-mycorrhizal fungi in germination of the mycoheterotrophic orchid *Pogoniopsis schenckii* Cogn. Front. Plant Sci. 10:1589. 10.3389/fpls.2019.01589, PMID: 31850049 PMC6896934

[ref197] Smith S. E. Read D. J. (2008). Mycorrhizal Symbiosis. Cambridge, UK: Academic Press.

[ref198] Spencer S. J. Tamminen M. V. Preheim S. P. Guo M. T. Briggs A. W. Brito I. L.. (2016). Massively parallel sequencing of single cells by epicPCR links functional genes with phylogenetic markers. ISME J. 10, 427–436. 10.1038/ismej.2015.124, PMID: 26394010 PMC4737934

[ref199] Stark C. Babik W. Durka W. (2009). Fungi from the roots of the common terrestrial orchid *Gymnadenia conopsea*. Mycol. Res. 113, 952–959. 10.1016/j.mycres.2009.05.002, PMID: 19486943

[ref200] Suárez J. P. Eguiguren J. S. Herrera P. Jost L. (2016). Do mycorrhizal fungi drive speciation in *Teagueia* (Orchidaceae) in the upper Pastaza watershed of Ecuador? Symbiosis 69, 161–168. 10.1007/s13199-016-0399-6

[ref201] Suárez J. P. Kottke I. (2016). Main fungal partners and different levels of specificity of orchid mycorrhizae in the tropical mountain forests of Ecuador. Lankesteriana 16, 299–305. 10.15517/lank.v16i2.26014

[ref202] Suárez J. P. Weiß M. Abele A. Garnica S. Oberwinkler F. Kottke I. (2009). “Epiphytic orchids in a mountain rain forest in southern Ecuador harbor groups of mycorrhiza-forming Tulasnellales and Sebacinales subgroup B (Basidiomycota)” in *Proceedings of the Second Scientific Conference on Andean Orchids*. eds. Pridgeon A. M. Suárez J. P.; January 2009 (Loja: Universidad Técnica Particular de Loja), 184–196.

[ref203] Sudheep N. M. Sridhar K. R. (2012). Non-mycorrhizal fungal endophytes in two orchids of Kaiga forest (Western Ghats), India. J. For. Res. 23, 453–460. 10.1007/s11676-012-0284-y

[ref204] Suetsugu K. Yamato M. Miura C. Yamaguchi K. Takahashi K. Ida Y.. (2017). Comparison of green and albino individuals of the partially mycoheterotrophic orchid *Epipactis helleborine* on molecular identities of mycorrhizal fungi, nutritional modes and gene expression in mycorrhizal roots. Mol. Ecol. 26, 1652–1669. 10.1111/mec.14021, PMID: 28099773

[ref205] Sufaati S. Agustini V. Suharno S. (2016). Short communication: *Fusarium* as endophyte of some terrestrial orchid from Papua, Indonesia. Biodiversitas 17, 366–371. 10.13057/biodiv/d170149

[ref206] Swarts N. D. Sinclair E. A. Francis A. Dixon K. W. (2010). Ecological specialization in mycorrhizal symbiosis leads to rarity in an endangered orchid. Mol. Ecol. 19, 3226–3242. 10.1111/j.1365-294X.2010.04736.x, PMID: 20618899

[ref207] Swift S. Munroe S. Im C. Tipton L. Hynson N. A. (2019). Remote tropical island colonization does not preclude symbiotic specialists: new evidence of mycorrhizal specificity across the geographic distribution of the Hawaiian endemic orchid *Anoectochilus sandvicensis*. Ann. Bot. 123, 657–666. 10.1093/aob/mcy198, PMID: 30380004 PMC6417469

[ref208] Tang L. (2020). Exploring microbial worlds. Nat. Methods 17:22. 10.1038/s41592-019-0707-1, PMID: 31907487

[ref209] Tao G. Liu Z. Y. Liu F. Gao Y. H. Cai L. (2013). Endophytic *Colletotrichum* species from *Bletilla ochracea* (Orchidaceae), with descriptions of seven new speices. Fungal Divers. 61, 139–164. 10.1007/s13225-013-0254-5

[ref210] Taylor D. L. (1997). The evolution of myco-heterotrophy and specificity in some North American orchids. dissertation. Berkeley: University of California.

[ref211] Taylor D. L. Bruns T. D. (1999). Population, habitat and genetic correlates of mycorrhizal specialization in the ‘cheating’ orchids *Corallorhiza maculata* and *C. mertensiana*. Mol. Ecol. 8, 1719–1732. 10.1046/j.1365-294x.1999.00760.x, PMID: 10583834

[ref212] Taylor D. L. Bruns T. D. Leake J. R. Read D. J. (2002). “Mycorrhizal specificity and function in myco-heterotrophic plants,” in Mycorrhizal Ecology: Ecological Studies, eds. van der Heijden M. G. A. Sanders I. R. (Berlin, Heidelberg: Springer), 375–414.

[ref213] Taylor D. L. Herriott I. C. Stone K. E. McFarland J. W. Booth M. G. Leigh M. B. (2010). Structure and resilience of fungal communities in Alaskan boreal forest soils. Can. J. For. Res. 40, 1288–1301. 10.1139/X10-081

[ref214] Taylor D. L. McCormick M. K. (2008). Internal transcribed spacer primers and sequences for improved characterization of basidiomycetous orchid mycorrhizas. New Phytol. 177, 1020–1033. 10.1111/j.1469-8137.2007.02320.x, PMID: 18086221

[ref215] Tedersoo L. Bahram M. (2019). Mycorrhizal types differ in ecophysiology and alter plant nutrition and soil processes. Biol. Rev. 94, 1857–1880. 10.1111/brv.12538, PMID: 31270944

[ref216] Tedersoo L. Bahram M. Zobel M. (2020). How mycorrhizal associations drive plant population and community biology. Science 367:eaba1223. 10.1126/science.aba1223, PMID: 32079744

[ref217] Tedersoo L. Lindahl B. (2016). Fungal identification biases in microbiome projects. Environ. Microbiol. Rep. 8, 774–779. 10.1111/1758-2229.12438, PMID: 27348848

[ref218] Tedersoo L. Nilsson R. H. (2016). “Molecular identification of fungi,” in Molecular Mycorrhizal Symbiosis. ed. Martin F. (Hoboken, NJ: John Wiley and Sons), 299–322.

[ref219] Tedersoo L. Pärtel K. Jairus T. Gates G. Põldmaa K. Tamm H. (2009). Ascomycetes associated with ectomycorrhizas: molecular diversity and ecology with particular reference to the Helotiales. Environ. Microbiol. 11, 3166–3178. 10.1111/j.1462-2920.2009.02020.x, PMID: 19671076

[ref220] Tedersoo L. Tooming-Klunderud A. Anslan S. (2018). PacBio metabarcoding of Fungi and other eukaryotes: errors, biases and perspectives. New Phytol. 217, 1370–1385. 10.1111/nph.14776, PMID: 28906012

[ref221] Těšitelová T. Jersáková J. Roy M. Kubátová B. Těšitel J. Urfus T.. (2013). Ploidy-specific symbiotic interactions: divergence of mycorrhizal fungi between cytotypes of the *Gymnadenia conopsea* group (Orchidaceae). New Phytol. 199, 1022–1033. 10.1111/nph.12348, PMID: 23731358

[ref222] Těšitelová T. Kotilínek M. Jersáková J. Joly F. X. Košnar J. Tatarenko I.. (2015). Two widespread green *Neottia* species (Orchidaceae) show mycorrhizal preference for Sebacinales in various habitats and ontogenetic stages. Mol. Ecol. 24, 1122–1134. 10.1111/mec.13088, PMID: 25612936

[ref223] Těšitelová T. Těšitel J. Jersáková J. Říhová G. Selosse M.-A. (2012). Symbiotic germination capability of four *Epipactis* species (Orchidaceae) is broader than expected from adult ecology. Am. J. Bot. 99, 1020–1032. 10.3732/ajb.1100503, PMID: 22688426

[ref224] The Plant List (2013). Version 1.1. Published on the Internet. Available at: http://www.theplantlist.org/ (Accessed April 1, 2020).

[ref225] Thixton H. L. Esselman E. J. Corey L. L. Zettler L. W. (2020). Further evidence of *Ceratobasidium* D.P. Rogers (Basidiomycota) serving as the ubiquitous fungal associate of *Platanthera leucophaea* (Orchidaceae) in the north American tallgrass prairie. Bot. Stud. 61:12. 10.1186/s40529-020-00289-z, PMID: 32297130 PMC7158956

[ref226] Tilman D. (1982). Resource Competition and Community Structure. Princeton, NJ: Princeton University Press. 7162524

[ref227] Toju H. Vannette R. L. Gauthier M. P. L. Dhami M. K. Fukami T. (2018). Priority effects can persist across floral generations in nectar microbial metacommunities. Oikos 127, 345–352. 10.1111/oik.04243

[ref228] Toussaint A. Bueno G. Davison J. Moora M. Tedersoo L. Zobel M.. (2020). Asymmetric patterns of global diversity among plants and mycorrhizal fungi. J. Veg. Sci. 31, 355–366. 10.1111/jvs.12837

[ref229] Tremblay R. L. Ackerman J. D. Zimmerman J. K. Calvo R. N. (2005). Variation in sexual reproduction in orchids and its evolutionary consequences: a spasmodic journey to diversification. Biol. J. Linn. Soc. 84, 1–54. 10.1111/j.1095-8312.2004.00400.x

[ref230] Unruh S. A. Chris Pires J. Zettler L. Erba L. Grigoriev I. Barry K.. (2019). Shallow genome sequencing for phylogenomics of mycorrhizal fungi from endangered orchids. BioRxiv [Preprint]. 10.1101/862763

[ref232] Vandeputte D. Kathagen G. D’hoe K. Vieira-Silva S. Valles-Colomer M. Sabino J.. (2017). Quantitative microbiome profiling links gut community variation to microbial load. Nature 551, 507–511. 10.1038/nature24460, PMID: 29143816

[ref231] van der Heijden M. G. A. Martin F. M. Selosse M.-A. Sanders I. R. (2015). Mycorrhizal ecology and evolution: the past, the present, and the future. New Phytol. 205, 1406–1423. 10.1111/nph.13288, PMID: 25639293

[ref233] Vellend M. (2016). The Theory of Ecological Communities. Princeton, NJ: Princeton University Press.

[ref234] Vogt-Schilb H. Těšitelová T. Kotilínek M. Sucháček P. Kohout P. Jersáková J. (2020). Altered rhizoctonia assemblages in grasslands on ex-arable land support germination of mycorrhizal generalist, not specialist orchids. New Phytol. 227, 1200–1212. 10.1111/nph.16604, PMID: 32285948

[ref235] Voyron S. Ercole E. Ghignone S. Perotto S. Girlanda M. (2017). Fine-scale spatial distribution of orchid mycorrhizal fungi in the soil of host-rich grasslands. New Phytol. 213, 1428–1439. 10.1111/nph.14286, PMID: 27861936

[ref236] Vujanovic V. St-Arnaud M. Barabé D. Thibeault G. (2000). Viability testing of orchid seed and the promotion of colouration and germination. Ann. Bot. 86, 79–86. 10.1006/anbo.2000.1162

[ref237] Wang X. M. Li Y. J. Song X. Q. Meng Q. W. Zhu J. Zhao Y.. (2017). Influence of host tree species on isolation and communities of mycorrhizal and endophytic fungi from roots of a tropical epiphytic orchid, *Dendrobium sinense* (Orchidaceae). Mycorrhiza 27, 709–718. 10.1007/s00572-017-0787-7, PMID: 28685256

[ref238] Waterman R. J. Bidartondo M. I. Stofberg J. Combs J. K. Gebauer G. Savolainen V.. (2011). The effects of above-and belowground mutualisms on orchid speciation and coexistence. Am. Nat. 177, E54–E68. 10.1086/657955, PMID: 21460551

[ref239] Waud M. Brys R. Van Landuyt W. Lievens B. Jacquemyn H. (2017). Mycorrhizal specificity does not limit the distribution of an endangered orchid species. Mol. Ecol. 26, 1687–1701. 10.1111/mec.14014, PMID: 28100022

[ref240] Waud M. Busschaert P. Lievens B. Jacquemyn H. (2016a). Specificity and localised distribution of mycorrhizal fungi in the soil may contribute to co-existence of orchid species. Fungal Ecol. 20, 155–165. 10.1016/j.funeco.2015.12.008

[ref241] Waud M. Busschaert P. Ruyters S. Jacquemyn H. Lievens B. (2014). Impact of primer choice on characterization of orchid mycorrhizal communities using 454 pyrosequencing. Mol. Ecol. Resour. 14, 679–699. 10.1111/1755-0998.12229, PMID: 24460947

[ref242] Waud M. Wiegand T. Brys R. Lievens B. Jacquemyn H. (2016b). Nonrandom seedling establishment corresponds with distance-dependent decline in mycorrhizal abundance in two terrestrial orchids. New Phytol. 211, 255–264. 10.1111/nph.13894, PMID: 26876007

[ref243] Weiß M. Waller F. Zuccaro A. Selosse M.-A. (2016). Sebacinales—one thousand and one interactions with land plants. New Phytol. 211, 20–40. 10.1111/nph.13977, PMID: 27193559

[ref244] Xing X. K. Gai X. G. Liu Q. Hart M. M. Guo S. X. (2015). Mycorrhizal fungal diversity and community composition in a lithophytic and epiphytic orchid. Mycorrhiza 25, 289–296. 10.1007/s00572-014-0612-5, PMID: 25319065

[ref245] Xing X. K. Gao Y. Zhao Z. Y. Waud M. Duffy K. J. Selosse M.-A.. (2020). Similarity in mycorrhizal communities associating with two widespread terrestrial orchids decays with distance. J. Biogeogr. 47, 421–433. 10.1111/jbi.13728

[ref246] Xing X. K. Jacquemyn H. Gai X. G. Gao Y. Liu Q. Zhao Z. Y.. (2019). The impact of life form on the architecture of orchid mycorrhizal networks in tropical forest. Oikos 128, 1254–1264. 10.1111/oik.06363

[ref247] Xing X. K. Ma X. T. Men J. X. Chen Y. H. Guo S. X. (2017). Phylogenetic constrains on mycorrhizal specificity in eight *Dendrobium* (Orchidaceae) species. Sci. China Life Sci. 60, 536–544. 10.1007/s11427-017-9020-1, PMID: 28299575

[ref248] Xu J. T. Guo S. X. (1989). Fungus associated with nutrition of seed germination of *Gastrodia elata*—*Mycena osmundicola* Lange. Acta Mycol. Sin. 8, 221–226. 10.13346/j.mycosystema.1989.03.011

[ref249] Xun W. B. Li W. Xiong W. Ren Y. Liu Y. P. Miao Y. Z.. (2019). Diversity-triggered deterministic bacterial assembly constrains community functions. Nat. Commun. 10:3833. 10.1038/s41467-019-11787-5, PMID: 31444343 PMC6707308

[ref250] Yagame T. Funabiki E. Nagasawa E. Fukiharu T. Iwase K. (2013). Identification and symbiotic ability of Psathyrellaceae fungi isolated from a photosynthetic orchid, *Cremastra appendiculata* (Orchidaceae). Am. J. Bot. 100, 1823–1830. 10.3732/ajb.1300099, PMID: 24026354

[ref251] Yagame T. Orihara T. Selosse M.-A. Yamato M. Iwase K. (2012). Mixotrophy of *Platanthera minor*, an orchid associated with ectomycorrhiza-forming Ceratobasidiaceae fungi. New Phytol. 193, 178–187. 10.1111/j.1469-8137.2011.03896.x, PMID: 21995447

[ref252] Zeng X. H. Diao H. X. Ni Z. Y. Shao L. Jiang K. Hu C.. (2021). Temporal variation in community composition of root associated endophytic fungi and carbon and nitrogen stable isotope abundance in two *Bletilla* species (Orchidaceae). Plan. Theory 10, 1–18. 10.3390/plants10010018, PMID: 33374219 PMC7824424

[ref253] Zettler L. W. Corey L. L. (2018). “Orchid mycorrhizal fungi: isolation and identification techniques,” in Orchid Propagation: From Laboratories to Greenhouses—Methods and Protocols. eds. Lee Y. I. Yeung E. C. (New York, NY: Humana Press), 27–59.

[ref254] Zettler L. W. Piskin K. A. Stewart S. L. Hartsock J. Bowles M. L. Bell T. J. (2005). Protocorm mycobionts of the federally threatened eastern prairie fringed orchid, *Platanthera leucophaea* (Nutt.) Lindley, and a technique to prompt leaf elongation in seedlings. Stud. Mycol. 53, 163–171. 10.3114/sim.53.1.163

[ref255] Zhang L. C. Chen J. Lv Y. L. Gao C. Guo S. X. (2012). *Mycena* sp., a mycorrhizal fungus of the orchid *Dendrobium officinale*. Mycol. Prog. 11, 395–401. 10.1007/s11557-011-0754-1

[ref256] Zhang Y. B. Du H. D. Jin X. H. Ma K. P. (2015). Species diversity and geographic distribution of wild Orchidaceae in China. Chin. Sci. Bull. 60, 179–188. 10.1360/N972014-00480, PMID: 26555336

[ref257] Zhang Y. Li Y. Y. Chen X. M. Guo S. X. Lee Y. I. (2020). Effect of different mycobionts on symbiotic germination and seedling growth of *Dendrobium officinale*, an important medicinal orchid. Bot. Stud. 61:2. 10.1186/s40529-019-0278-6, PMID: 31989371 PMC6985412

[ref258] Zhang G. Q. Liu K. W. Li Z. Lohaus R. Hsiao Y. Y. Niu S. C.. (2017). The *Apostasia* genome and the evolution of orchids. Nature 549, 379–383. 10.1038/nature23897, PMID: 28902843 PMC7416622

[ref259] Zhang G. Q. Xu Q. Bian C. Tsai W. C. Yeh C. M. Liu K. W.. (2016). The *Dendrobium catenatum* Lindl. genome sequence provides insights into polysaccharide synthase, floral development and adaptive evolution. Sci. Rep. 6:19029. 10.1038/srep19029, PMID: 26754549 PMC4709516

[ref260] Zi X. M. Sheng C. L. Goodale U. M. Shao S. C. Gao J. Y. (2014). In situ seed baiting to isolate germination-enhancing fungi for an epiphytic orchid, *Dendrobium aphyllum* (Orchidaceae). Mycorrhiza 24, 487–499. 10.1007/s00572-014-0565-8, PMID: 24563211

[ref261] Zimmer K. Meyer C. Gebauer G. (2008). The ectomycorrhizal specialist orchid *Corallorhiza trifida* is a partial myco-heterotroph. New Phytol. 178, 395–400. 10.1111/j.1469-8137.2007.02362.x, PMID: 18221248

