# Supplementary material for: Progress and Prospects of Mycorrhizal Fungal Diversity in Orchids
Source: Front Plant Sci. 2021 May 7;12:646325. doi: 10.3389/fpls.2021.646325 (PMC8138444; doi:10.3389/fpls.2021.646325)
Supplement: Supplementary file 1 [file Data_Sheet_1.pdf]

Supplementary Information

Review title: Progress and Prospects of Mycorrhizal Fungi Diversity in Orchids

Authors: *Taiqiang Li, Wenke Yang, Shimao Wu, Marc-André Selosse and Jiangyun Gao* *

Review acceptance date: **12 April 2021**

The following Supplementary Information is available for this article:

Table S1 | Recommended primers for high-throughput sequencing of orchid mycorrhizal partners.

Primer name	Sequence (5'-3' direction)	Reference
Modified ITS1ngs	TCCGTAGGTGAACCTGC	Oja *et al.* (2015)
Modified ITS1Fngs	GGTCATTTAGAGGAAGTAA	Oja *et al.* (2015)
ITS1	TCCGTAGGTGAACCTGCGG	White *et al.* (1990)
Modified ITS4ngs	TCCTSCGCTTATTGATATGC	Oja *et al.* (2015)
ITS4Tul2	TTCTTTTCCTCCGCTGAWTA	Oja *et al.* (2015)
ITS1F	CTTGGTCATTTAGAGGAAGTAA	Gardes and Bruns (1993)
ITS86F	GTGAATCATCGAATCTTTGAA	Turenne *et al.* (1999)
ITS4	TCCTCCGCTTATTGATATGC	White *et al.* (1990)
ITS4Tul	CCGCCAGATTCACACATTGA	Taylor (1997)
TW14ngs	CTATCCTGRGRGAAAYTTC	Tedersoo *et al.* (2014)

Oja, J., Kohout, P., Tedersoo, L., Kull, T., and Kõljalg, U. (2015). Temporal patterns of orchid mycorrhizal fungi in meadows and forests as revealed by 454 pyrosequencing. *New Phytol.* **205**, 1608-1618. doi: 10.1111/nph.13223

White, T. J., Bruns, T., Lee, S., and Taylor, J. (1990). "Amplification and direct sequencing of fungal ribosomal RNA genes for phylogenetics," in PCR Protocols: A Guide to Methods and Applications, **eds. M. A. Innis, D. H. Gelfand, J. J. Sninsky, and T. J. White** (San Diego, CA, USA: Academic Press), 315-322.

Gardes, M., and Bruns, T. D. (1993). ITS primers with enhanced specificity for basidiomycetes-application to the identification of mycorrhizae and rusts. *Mol. Ecol.* **2**, 113-118. doi: 10.1111/j.1365-294X.1993.tb00005.x

Turenne, C. Y., Sanche, S. E., Hoban, D. J., Karlowsky, J. A., Kabani, A. M. (1999). Rapid identification of fungi by using the ITS2 genetic region and an automated fluorescent capillary electrophoresis system. *J. Clin. Microbiol.* **37**, 1846–1851. doi: 10.1128/JCM

Taylor, D. L. (1997). The evolution of myco-heterotrophy and specificity in some North American orchids. dissertation. Berkeley: University of California.

Tedersoo, L., Bahram, M., Põlme, S., Kõljalg, U., Yorou, N. S., Wijesundera, R., et al. (2014). Global diversity and geography of soil fungi. *Science* **346**:1256688. doi: 10.1126/science.1256688

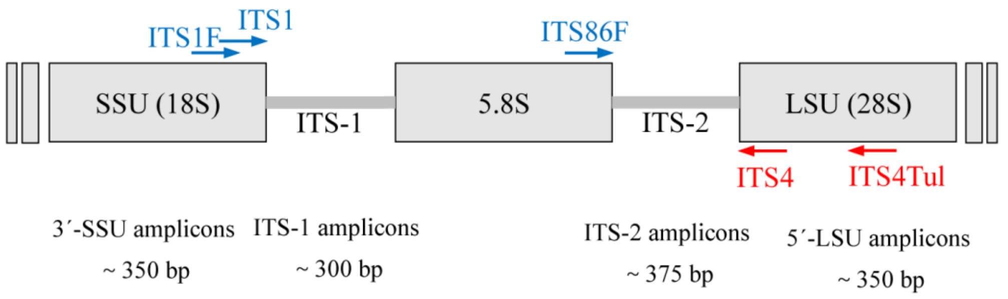

Figure S1 | Schematic representation of primers recommended for MiSeq PE300 and HiSeq PE250 amplicon sequencing. Blue for forward primer, red for reverse primer.
